# Spatial architecture of high-grade glioma reveals tumor heterogeneity within distinct domains

**DOI:** 10.1093/noajnl/vdad142

**Published:** 2023-11-01

**Authors:** Joel J D Moffet, Oluwaseun E Fatunla, Lutz Freytag, Jurgen Kriel, Jordan J Jones, Samuel J Roberts-Thomson, Anna Pavenko, David K Scoville, Liang Zhang, Yan Liang, Andrew P Morokoff, James R Whittle, Saskia Freytag, Sarah A Best

**Affiliations:** Personalised Oncology Division, The Walter and Eliza Hall Institute of Medical Research, Melbourne, Victoria, Australia; Department of Medical Biology, University of Melbourne, Melbourne, Victoria, Australia; Personalised Oncology Division, The Walter and Eliza Hall Institute of Medical Research, Melbourne, Victoria, Australia; Department of Medical Biology, University of Melbourne, Melbourne, Victoria, Australia; Personalised Oncology Division, The Walter and Eliza Hall Institute of Medical Research, Melbourne, Victoria, Australia; Personalised Oncology Division, The Walter and Eliza Hall Institute of Medical Research, Melbourne, Victoria, Australia; Department of Medical Biology, University of Melbourne, Melbourne, Victoria, Australia; Department of Surgery, Royal Melbourne Hospital, Melbourne, Victoria, Australia; Department of Anatomical Pathology, Royal Melbourne Hospital, Melbourne, Victoria, Australia; NanoString Technologies Inc., Seattle, Washington, USA; NanoString Technologies Inc., Seattle, Washington, USA; NanoString Technologies Inc., Seattle, Washington, USA; NanoString Technologies Inc., Seattle, Washington, USA; Department of Surgery, Royal Melbourne Hospital, Melbourne, Victoria, Australia; Personalised Oncology Division, The Walter and Eliza Hall Institute of Medical Research, Melbourne, Victoria, Australia; Department of Medical Biology, University of Melbourne, Melbourne, Victoria, Australia; Department of Medical Oncology, Peter MacCallum Cancer Centre, Melbourne, Victoria, Australia; Personalised Oncology Division, The Walter and Eliza Hall Institute of Medical Research, Melbourne, Victoria, Australia; Department of Medical Biology, University of Melbourne, Melbourne, Victoria, Australia; Personalised Oncology Division, The Walter and Eliza Hall Institute of Medical Research, Melbourne, Victoria, Australia; Department of Medical Biology, University of Melbourne, Melbourne, Victoria, Australia

**Keywords:** brain cancer, glioma, spatial transcriptomics, sequencing, immune microenvironment

## Abstract

**Background:**

High-grade gliomas (HGGs) are aggressive primary brain cancers with poor response to standard regimens, driven by immense heterogeneity. In isocitrate dehydrogenase (*IDH*) wild-type HGG (glioblastoma, GBM), increased intratumoral heterogeneity is associated with more aggressive disease.

**Methods:**

Spatial technologies can dissect complex heterogeneity within the tumor ecosystem by preserving cellular organization *in situ*. We employed GeoMx digital spatial profiling, CosMx spatial molecular imaging, Xenium *in situ* mapping and Visium spatial gene expression in experimental and validation patient cohorts to interrogate the transcriptional landscape in HGG.

**Results:**

Here, we construct a high-resolution molecular map of heterogeneity in GBM and *IDH*-mutant patient samples to investigate the cellular communities that compose HGG. We uncovered striking diversity in the tumor landscape and degree of spatial heterogeneity within the cellular composition of the tumors. The immune distribution was diverse between samples, however, consistently correlated spatially with distinct tumor cell phenotypes, validated across tumor cohorts. Reconstructing the tumor architecture revealed two distinct niches, one composed of tumor cells that most closely resemble normal glial cells, associated with microglia, and the other niche populated by monocytes and mesenchymal tumor cells.

**Conclusions:**

This primary study reveals high levels of intratumoral heterogeneity in HGGs, associated with a diverse immune landscape within spatially localized regions.

## Graphical Abstract

**Graphical Abstract:**
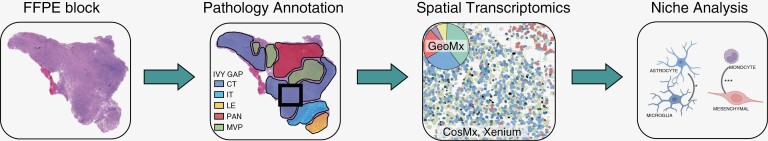

**Key Points**

1. Increased inter- and intratumoral heterogeneity in glioblastoma tumors.
2. Intratumoral heterogeneity is detected between cycling and noncycling tumor cells.
3. Tumor cells are organized in distinct domains with associated immune landscapes.

**Importance of the Study**

Integrating digital spatial profiling and spatial molecular imaging, this study reveals immense spatial heterogeneity in *IDH1*-wt glioblastoma (GBM) samples compared to *IDH1*-mutant tumors, characterized by the presence of the mesenchymal tumor cell lineage. Through the isolation and analysis of cycling tumor cells, we find that progenitor cells are distinctly identified within this subset, highlighting the plasticity of glioma cell states. Analysis of the immune compartment revealed discrete spatial communities with patterns of tumor and immune cell interaction that validated across GBM cohorts and independent spatial sequencing technologies. This discretization suggests success of immunotherapeutic targeting strategies could differ across regions depending on the local tumor microenvironment. Hence, understanding the localized interplay between different tumor cell states and immune cell types will be critical in advancing therapeutics.

High-grade gliomas (HGGs), characterized as World Health Organization (WHO) grades 3 and 4, are aggressive forms of brain cancer with poor survival outcomes and treatment regimens that have not changed in decades. The evolution of WHO grading^1^ has increasingly incorporated molecular analysis that stratifies glioma based on isocitrate dehydrogenase (*IDH*) status into groups with distinct biology and clinical outcomes with mutations in *IDH1/2* predicting improved survival (5–15 years, depending on grade).^2–5^*IDH-*mutated gliomas are further stratified into oligodendroglioma, which is defined by the presence of 1p19q co-deletion and astrocytoma (1p19q intact). *IDH* wild-type status characterizes grade 4 glioblastoma (GBM) with a median prognosis with treatment of just 13–18 months.^6^ Multiple genetic alterations are additionally used to classify gliomas, including *ATRX* loss, deletions or inactivating mutations in *TP53, PTEN*, *NF1*, and *CDKN2A/B*, amplification of *EGFR*, *PDGFRA*, and *CDK4/6,* and gain of chromosome 7 and loss of 10.^1,7^ While molecular profiling provides important prognostic value, novel therapies that target the molecular drivers of HGG have yet to show clinical benefit.^8,9^ Thus, patients still receive radiation and alkylating chemotherapy^4,5,10,11^ using regimens unchallenged for the past 25 years, highlighting a need for improved stratification and treatment options.

In addition to genetic alterations, intrinsic cellular plasticity adds to the intra- and interpatient phenotypic variability observed in glioma.^12^ Advances in single-cell sequencing revealed that tumor cells exhibit transcriptional signatures mirroring the early development of the healthy human brain, including astrocytic, oligodendrocytic, neural, mesenchymal, and highly proliferative progenitor cell states.^13–17^*IDH*-mutant HGG displays a bi-lineage hierarchy of astrocytic and oligodendrocytic cell states, with a rare subpopulation of progenitor cells,^16,17^ defining astrocytoma and oligodendroglioma tumors. In comparison to *IDH*-mutant HGG, GBM tumors are characterized by greater intratumoral heterogeneity, the extent correlating with survival,^18^ resulting in a mixture of each cell state within the tumor.^13,14,18^ Upon recurrence, there is a shift toward the mesenchymal lineage likely induced by immune cells and interferon signaling.^19–21^ The immense plasticity of glioma tumor cells allows them to reprogram and evade treatment, contributing to the poor survival of patients.^22^

Overcoming tumor heterogeneity is a major challenge for cancer treatment, with the past decade focusing on novel immunotherapies that drive an antigen-mediated antitumor response, determined by the composition and extent of immune infiltration. While these strategies have been successful in many solid cancers, particularly those with high tumor mutational burden, to date, efficacy in glioma has been limited. A number of factors likely contribute to the lack of benefit in glioma. First, the immune microenvironment of the brain is classically associated with poor infiltration, with an immunosuppressive environment and largely composed of resident microglia cells.^23^ Indeed, both *IDH*-mutant high-grade and GBM tumors are dominated by myeloid cells, though GBM tumors exhibit increased bone-marrow-derived macrophages (BDM) and lymphocytes compared to *IDH*-mutant tumors.^24^ However, there have been reported association of tumor cell states and specific immune cell infiltrate, such as mesenchymal cells with myeloid^14,20^ and T-cell infiltration,^25^ which were associated with a more unfavorable prognosis.^20^ Tumor cells together with the immune microenvironment create a complex milieu that ultimately promotes adaptability and disease progression, highlighting the need for a detailed understanding of the interactions between immune cells and different tumor cell states.

To capture a deeper understanding of the tumor microenvironment of HGGs, we perform a combination of digital spatial profiling,^26^ spatial molecular imaging,^27^*in situ* mapping^28^ and spatial gene expression^15^ in experimental and validation patient cohorts. In concert, these technologies, which preserve the tissue architecture, uncover the overall identity of the spatial environment^29^ with complementary strengths. Sequencing-based technologies capture regions of tissue to provide next-generation sequencing output of the whole transcriptome, and while not providing single-cell resolution, these technologies are not limited by gene number. In contrast, high-plex imaging technologies using a probe-based readout offer subcellular resolution, but are currently limited to a restricted number of gene targets. Integrating these technologies, we confirm immense spatial heterogeneity in GBM samples compared to *IDH1*-mutant tumors, characterized by the presence of the mesenchymal tumor cell lineage. Through the isolation and analysis of cycling Ki67^+^ tumor cells, we find that progenitor cells emerge within this subset, highlighting the plasticity of glioma cell states. Analysis of the immune compartment revealed discrete neighborhoods with patterns of tumor and immune cell interaction that validated across GBM cohorts and independent spatial sequencing technologies. Finally, we create a map of tumor cell state and immune cell interaction in the GBM microenvironment. Our findings highlight the immense heterogeneity of tumor cell states in GBM and their relationship with the immune microenvironment.

## Materials and Methods

### Clinical Samples

All tissue samples were obtained from the Royal Melbourne Hospital Neurosurgery Brain and Spine Tumour Tissue Bank (Melbourne Health Ethics #2020.214). Formalin-fixed paraffin-embedded tissues were sectioned at 5 μm thickness and mounted onto Superfrost Plus slides within 1 month of spatial transcriptomics analysis. Slides were stored at 4°C with a desiccator and shipped to NanoString (Seattle, WA) for Technology Access Program (TAP) processing for both GeoMx and CosMx analyses.

### Immunohistochemistry

Immunohistochemistry of GFAP (Dako Z0334; 1:50 dilution; epitope retrieval: Roche Ventana ULTRA Cell Conditioning Solution 1 pH 8.0-8.5 (CC1) 32 min at 36°C), IDH1-R132H (Dianova IDA-H09; 1:50 dilution; epitope retrieval: CC1 32 min at 100°C), CD45 (Dako M0701; 1:1000 dilution; epitope retrieval CC1 32 min at 100°C), KI67 (Dako M7240; 1:100 dilution; epitope retrieval: CC1 32 min at 100°C) and PDGFRA (Abcam ab203491; 1:200 dilution; epitope retrieval: EDTA buffer, pH 8.0–8.5 at 100°C for 15 min) was performed. Hematoxylin and eosin (H&E)-stained sections and immunohistochemistry slides were scanned using the 3D Histech Brightfield (20X) and processed using CaseCentre online software.

### Pathology Review

Pathology review was performed according to The Ivy Glioblastoma Atlas Project (Ivy GAP) classification^30^ (Methods Supplement).

### GeoMx Dataset

GeoMx data were generated and then preprocessed. Data were normalized using an upper-quartile normalization followed by batch correction for experimental batch and sex. Deconvolution was performed using the spatialDecon package (v1.6.0) with various reference datasets. To assess intra- and intertumoral heterogeneity of gene expression a Shannon-entropy approach was employed (for further details, see Methods Supplement).

### CosMx Dataset

CosMx data were preprocessed, including cell segmentation and normalization, and filtered to retain only cells with robust data. Cells were annotated using a hierarchical strategy combining manual and automated annotation. To identify spatial correlations between cell types/states, we performed modularity analysis and niche identification (for further details, see Methods Supplement).

### Xenium Dataset

Xenium data were preprocessed, including nuclei segmentation and normalization, and filtered to retain only nuclei with robust data. Nuclei were annotated using a hierarchical strategy combining manual and automated annotation. To identify spatial correlations between cell types/states, we performed modularity analysis and niche identification (for further details, see Methods Supplement).

### Visium Dataset

Processed data were downloaded and further annotations were obtained from the authors upon request.^15^ Co-location testing was performed using a permutation strategy (Methods Supplement).

### Data Availability

The GeoMx^®^ data are available at GEO accession GSE232469 and the CosMx and Xenium data are available at Mendeley Data DOI 10.17632/wc8tmdmsxm.2 and are publicly accessible.

### Code Availability

All code required for the analysis of the data can be found at https://github.com/SaskiaFreytag/spatial_brain_cancer/.

## Results

### Spatial Transcriptomics Analysis of High-Grade Glioma

**Figure 1.**
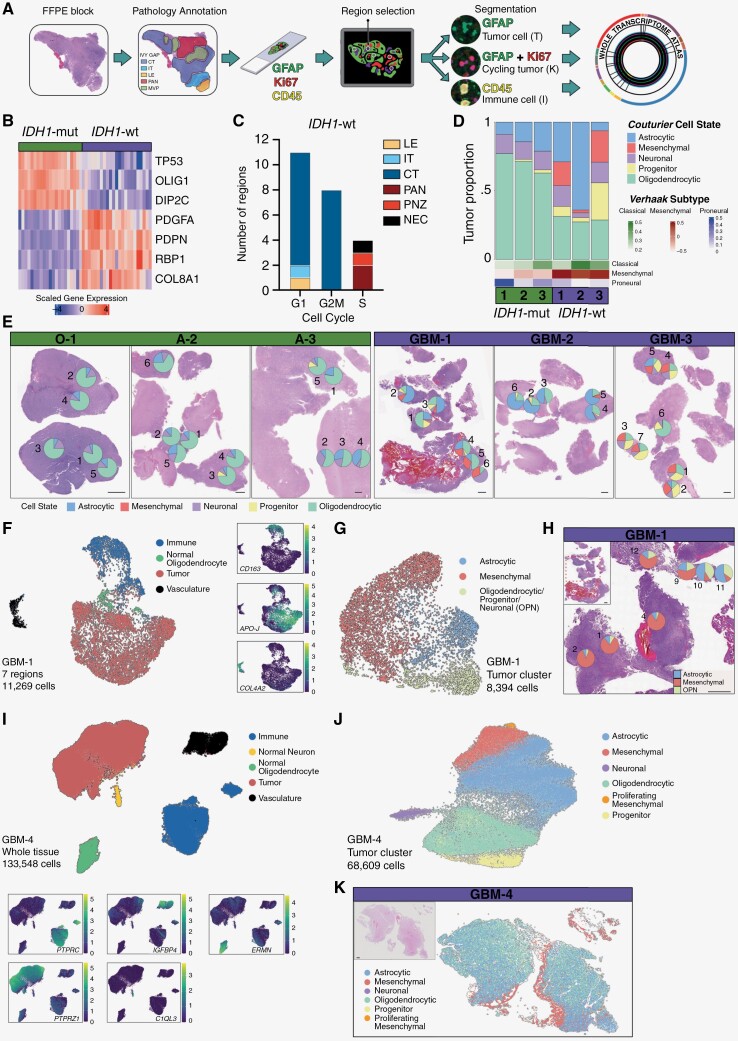
Detecting spatially heterogeneous regions in high-grade glioma. (A) Schematic of GeoMx digital spatial profiling workflow. Briefly, formalin-fixed paraffin-embedded (FFPE) blocks were sectioned and Ivy GAP pathology annotation performed to identify regions of interest. Slides were stained with GFAP, Ki67, and CD45 for segmentation into Tumor (GFAP^+^), proliferating tumor (GFAP^+^Ki67^+^), and immune (CD45^+^) samples, which were analyzed by whole transcriptome analysis. (B) Heat map of lineage-defining genes in *IDH1-*mut (*n* = 21 samples) and *IDH1-*wt (*n* = 23 samples) tumors. (C) Classification of *IDH1-*wt samples (*n* = 23) by Ivy GAP pathology annotation relative to cell cycling phase. (D) Deconvolution of tumor cell state proportions in tumor (T and K) samples for *IDH1-*mut (n = 3 patients) and *IDH1-*wt (*n* = 3 patients) tumors. (E) H&E of *IDH1-*mut and *IDH1-*wt GBM samples with tumor cell state proportions plotted over each region of interest. Scale, 1 mm. (F) UMAP plot depicting 11 269 cells identified in GBM-1 using CosMx separated into tumor, immune, normal glial, and vasculature cell types. Insets show UMAP colored by log expression of marker genes, *APO-J*, *CD163*, and *COL4A2*. (G) UMAP plot depicting 8394 tumor cells reclustered and colored by predicted tumor states based on *Couturier.*^13^ (H) H&E of GBM-1 sample with tumor cell state proportions calculated from single-cell transcriptomics. Scale, 1 mm. (I) UMAP plot depicting 133 548 cells identified in GBM-4 using Xenium separated into tumor, immune, vasculature, normal glial, and neuron cell types. Insets show UMAP colored by log expression of marker genes*, PTPRC*, *PTPRZ1*, *C1QL3*, *ERMN*, and *IGFBP4*. (J) UMAP plot depicting 68 609 tumor cells reclustered and colored by predicted tumor states based on *Couturier*^13^ and expression of marker genes. (K) H&E of GBM-4 sample with corresponding tumor cell annotation mapped in position. Scale, 1mm.

To interrogate the spatial intratumoral heterogeneity of HGG, we selected 3 *IDH* wild-type WHO grade 4 GBM (GBM −1 to 3) and 3 *IDH1-*mutant grade 3–4 oligodendroglioma (O-1) and astrocytoma (A-2, A-3) samples for spatial whole transcriptome analysis. Pathology review of the cases found these showed diagnostic morphological features, with astrocytoma and oligodendroglioma samples displaying moderately cellular tumors with predominantly pleomorphic cells, and glioblastomas demonstrating a higher degree of anaplasia and cellularity with hyperchromatic nuclei and irregular nuclear membrane (Table 1). Regions for spatial analysis were selected based on histological features, distribution of proliferating tumor cells and areas of central tumor (CT) bordering on infiltrating tumor and leading edge regions across the 6 samples (Figure 1A and Supplementary Figure 1A, B). Immunostaining for GFAP, CD45, and Ki67 protein expression enabled the segmentation and isolation of 34 segments representing tumor cells (T, GFAP^+^), 10 segments representing proliferating tumor cells (K, GFAP^+^Ki67^+^), and 20 segments representing immune cells (I, CD45^+^) using digital spatial profiling on the NanoString GeoMx platform (Table 2, Supplementary Tables S1 and S2).

**Table 1.** Characteristics of Patient Samples

Patient information				Clinical information								
Sample	Age	Sex	Tumor location	IDH1	ATRX	p53 (TP53)	p16 (CDKN2A)	Ki67	CD45	Cytology	WHO grade	Comments
O-1	35	F	Left frontal	Mutated	Wild type	Wild type	Positive	8%–13%	Sparse	O	3	Nil
A-2	27	F	Right parietal	Mutated	Mutated	Overexpressed	Positive	18%–23%	Moderate	A	3	Pretreated: radiation
A-3	45	M	Right frontal	Mutated	Mutated	Overexpressed	Negative	10%–15%	Moderate	A	4	Pretreated: radiation, temozolomide
GBM-1	67	M	Right temporo-occipital	Wild type	Wild type	Heterogeneous	Positive	10%– 15%	Sparse	GBM	4	MGMT methylated
GBM-2	49	M	Left frontal	Wild type	Wild type	Wild type	Negative	10%–15%	Dense	GBM	4	MGMT unmethylated
GBM-3	47	M	Left temporal	Wild type	Wild type	Heterogeneous	Negative	20%–30%	Dense	GBM	4	MGMT unmethylated
GBM-4	62	M	Right temporal	Wild type	Wild type	Wild type	Negative	60%	Moderate	GBM	4	MGMT methylated

IDH1, ATRX, p53 (TP53), p16 (CDKN2A), Ki67 and CD45 were assessed with immunohistochemistry. Heterogeneous staining: overexpressed and wild-type areas. GBM, glioblastoma; A, astrocytoma; O, oligodendroglioma, F, female; M, male.

**Table 2.** GeoMx Spatial Transcriptomics Key Features

Features		*IDH1*-mut	*IDH*-wt
Sample	Patients	3	3
	ROI	16	18
	AOI	32	39
	Excluded	6	1
AOI	Tumor	16	18
	Ki67+	5	5
	CD45	5	15

ROI, region of interest; AOI, area of illumination.

To initially compare *IDH1-*mutant and *IDH-*wt tumors, we investigated the tumor cell compartment (T and K samples). Principal component analysis stratified the tumor types (Supplementary Figure 1C, D) and as expected, *IDH1-*mutant tumors expressed increased levels of *OLIG1* while *IDH-*wt GBM tumors expressed *PDGFA* among other stratifying genes (Figure 1B). To evaluate the activity of tumor cells across the different regions, we investigated the cell cycle based on transcriptional signature in the *IDH-*wt GBM tumors, which consisted of regions with a variety of pathology classifications (Supplementary Figure 1B). Regions associated with necrosis (NEC, PAN, PNZ) were not cycling and associated with S Phase, while regions classified as cellular tumor (CT) were in cell-cycle phases G1 and active mitosis G2M (Figure 1C). Next, we combined the bulk transcriptomic data within each tumor sample (T and K samples) to investigate each tumor independently. To determine the proportions of each tumor cell state within these mini-bulk mixtures, we performed deconvolution analysis into the *Couturier* tumor cell states^13^ (Supplementary Figure 2A). This reveals large differences between *IDH1-*mutant and *IDH-*wt tumors; most notably the increase of mesenchymal (*P* < .001) and progenitor (*P* < .001) tumor cells in *IDH-*wt compared to *IDH1-*mutant (Figure 1D). We also compared the results of deconvolution into core tumor cell states using *Verhaak* classification.^20^ In contrast to the deconvolution result, all 3 GBM tumors were classified as largely mesenchymal. When applied to individual regions this difference between *Verhaak* classification and deconvolution into core tumor cell states persists (Supplementary Figure 2B). Similarly, deconvolution appeared to be more informative compared to the classification of individual regions according to *Garofano* subtyping.^31^ Further investigation of the tumor states within each region revealed spatial heterogeneity with significant increases in intratumoral heterogeneity in the *IDH-*wt samples compared to *IDH1-*mutant in the mesenchymal (variance test, *P* < .001) and progenitor (variance test, *P* < .001) tumor cell populations (Figure 1E).

To further dissect GBM-1 at subcellular resolution using the NanoString CosMx platform,^27^ 7 regions across the tumor were selected based on the location of initial GeoMx analysis to capture the heterogeneity at single-cell resolution (Supplementary Figure 2C). From the 1000 RNA probes, we calculated sufficient gene distribution to assign tumor cell states and immune cell types of interest for the study (Supplementary Figure 2D, Methods Supplement). A total of 11 269 cells were assigned, containing 1 797 037 total transcripts with a mean of 174.4 molecules expressed per cell. These cells were clustered and annotated by tumor, nonmalignant (normal) oligodendrocytes, vasculature, or immune cells (Figure 1F). The tumor cell compartment was reclustered, revealing mesenchymal cells, astrocytic cells, and a progenitor cluster comprising of oligodendrocytic, progenitor and neuronal (OPN) tumor cell states (Figure 1G). To examine the spatial distribution of these tumor cells, we calculated the overall composition in each region. This identified regions enriched with mesenchymal cells, in close proximity to those enriched in astrocytic and progenitor cells (Figure 1H), similar to the degree of spatial heterogeneity observed in the GeoMx study. To further explore the single-cell heterogeneity of GBM in an independent sample, we obtained a fourth GBM, GBM-4 (Table 1), and performed Xenium *in situ* spatial analysis on the entire tissue section. Analysis of the total 133 548 cells in the sample identified a vast tumor population, together with immune cells, vasculature and nonmalignant oligodendrocytes and neurons (Figure 1I). Reclustering and annotating the tumor cell compartment revealed the 5-key *Couturier* cell states in addition to a proliferating-mesenchymal population, which could be mapped back onto the tumor tissue (Figure 1J, K). Examining the tumor as a whole enables us to see distinct domains of heterogeneity and together, these studies using multiple technologies highlight the spatial heterogeneity of glioma, amplified in *IDH-*wt GBM tumors.

### Heterogeneity in Spatially Localized Tumor Regions

**Figure. 2.**
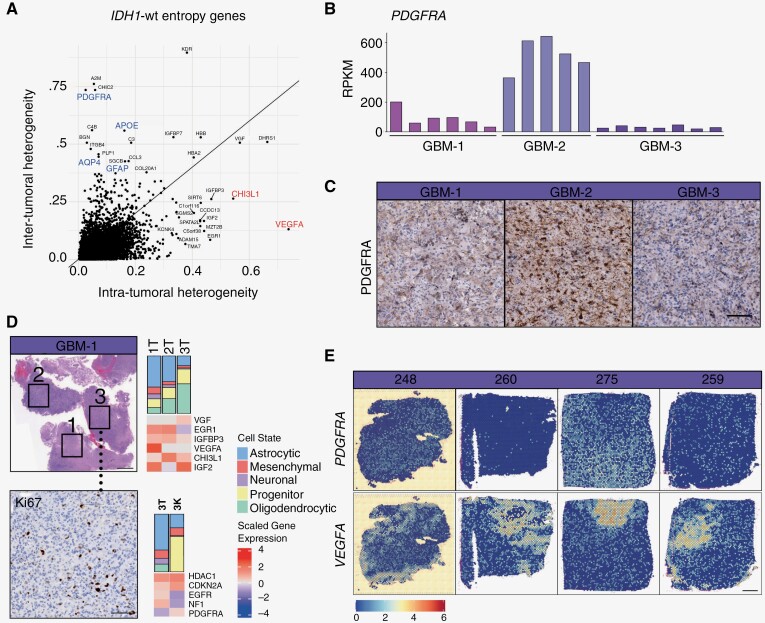
Enhanced spatial heterogeneity in *IDH1-*wt gliomas. (A) Shannon-entropy analysis of genes driving inter- and intratumoral heterogeneity in *IDH1-*wt GBM tumor samples (*n* = 3 patients). (B) *PDGFRA* gene expression in each GBM sample (*n* = 3). (C) Immunohistochemistry of PDGFR alpha expression in GBM samples. Scale, 100 µm. (D) GBM-1 spatial distribution of regions 1–3 (left) and associated tumor cell deconvolution and heat map of high-entropy genes in each sample (right). Region 3 (below) Ki67 immunostaining and GFAP^+^Ki67^+^ (K) and GFAP^+^Ki67^−^ (T) tumor cell deconvolution and heat map of high-entropy genes in each sample (right). (E) Expression of *PDGFRA* and *VEGFA* in representative GBM samples from *Ruiz-Moreno* Visium dataset.^14^

To examine the key genes driving inter- and intratumoral heterogeneity in *IDH-*wt tumors, we used a Shannon-entropy approach. Genes associated with the astrocytic (*APOE, AQP4, GFAP*) and oligodendrocytic (*PDGFRA*) lineages were found to drive heterogeneity between tumors and mesenchymal-associated genes (*CHI3L1, VEGFA*) were key in intratumoral heterogeneity (Figure 2A). The intertumoral heterogeneity observed in *PDGFRA* across patients was validated through RNA and protein expression (Figure 2B, C). Intratumoral heterogeneity was validated in GBM-1, where regions 1–3 in close proximity have widely varied proportions of astrocytic and oligodendrocytic tumor phenotypes and significant alterations in gene expression in *VEGFA*, among others (Figure 2D). Both spatial distribution and proliferation appear to play a role in intratumoral heterogeneity. Analysis of region 3 investigating cycling (K) and noncycling (T) tumor cells revealed immense cellular heterogeneity, especially in the progenitor tumor cell phenotype and *EGFR*, *NF1*, and *PDGFRA* gene expression. To validate genes associated with inter- and intratumoral heterogeneity in an independent dataset, we interrogated a GBM Visium spatial transcriptomics cohort.^15^ Similar to our *IDH-*wt cohort, *PDGFRA* expression was homogenous and varied between samples from 16 patients, whereas *VEGFA* expression displayed dynamic variability within patient samples (Figure 2E).

**Figure 3.**
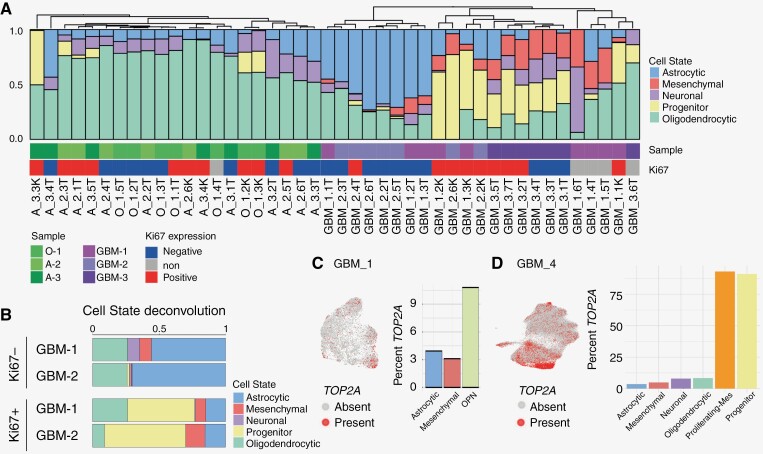
Dynamic cell states in proliferating tumor cells. (A) Deconvolution of tumor cell states in each *IDH1-*mut and *IDH1-*wt sample (T and K), with *MKI67* gene expression score for each sample. (B) Deconvolution of GBM-1 (*n* = 3 paired samples) and GBM-2 (*n* = 2 paired samples) Ki67^−^ vs Ki67^+^ sample in cellular tumor annotated regions. Proportions averaged across samples. GBM-3 not included due to no paired Ki67^+^ samples. (C) UMAP depicting presence of *TOP2A* expression in GBM-1 (left) and percentage of cells expressing *TOP2A* in each tumor state across all regions (right). (D) UMAP depicting presence of *TOP2A* expression in GBM-4 (left) and percentage of cells expressing *TOP2A* in each tumor state (right).

To further investigate the substantial cell phenotype differences between cycling and no-cycling tumor cells, we analyzed all T and K samples individually in *IDH1-*mutant and *IDH-*wt tumors (Figure 3A). Samples taken directly from Ki67^+^ cells (K), or bulk tumor samples with proliferation signatures (Ki67 positive), displayed altered tumor cell states compared to the nonproliferating counterparts. To better understand the impact on single tumors, GBM samples 1 and 2, each containing multiple-matched T and K samples, were separately investigated (Figure 3B). The progenitor cell state was significantly enriched in the cycling tumor compartment, compared to noncycling tumor cells (*P* < .001), at the expense of the astrocytic lineage (*P* < .05). To investigate this from the single-cell data, we mapped the expression of *TOP2A* across all cells in GBM-1 and GBM-4. As expected, the progenitor-containing OPN cluster was the most transcriptionally active, threefold higher than the astrocytic cell lineage in GBM-1 (Figure 3C), and the progenitor lineage and proliferating-mesenchymal cluster displayed the highest expression in GBM-4 (Figure 3D). This finding is consistent with the association of proliferation and the glioma stem cell with the progenitor subtype in previous studies.^13^

### Spatially Distinct Patterns of Immune Infiltration

**Figure 4.**
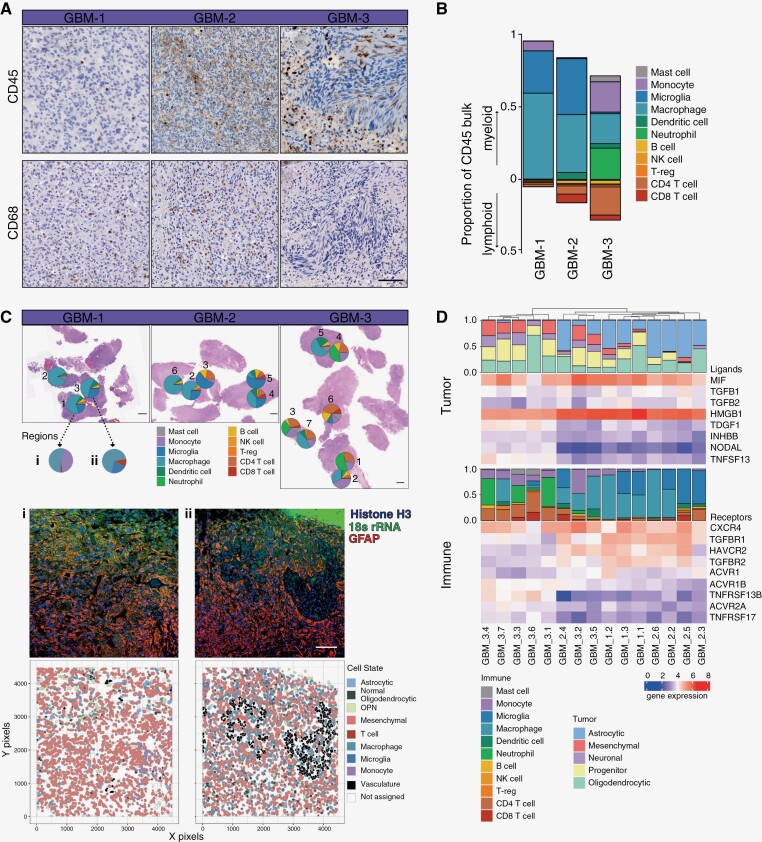
Immune infiltration in glioblastoma. (A) IHC staining of CD45 and CD68 protein expression aligning with regions for transcriptomics. Scale, 100 µm. (B) Average proportion of myeloid and lymphoid immune infiltrate in GBM samples, as determined from deconvolution. (C) H&E of GBM samples with immune proportions mapped spatially. Scale, 1 mm. (i, ii.) Corresponding regions analyzed by single-cell transcriptomics. Average immune proportions, immunofluorescence, and Voronoi plots of each annotated cell in frame. (D) Heat maps of tumor-expressing ligands and immune-expressing receptors with deconvolution of cell states.

The brain has a distinct immune composition within the tumor microenvironment, largely consisting of Tumor Associated Macrophages (TAMs)—microglia (MG) and BDM, with poor infiltration of lymphocytes.^23^ Investigation of the immune compartment across the *IDH-*wt GBM samples revealed variation in CD45^+^ infiltration, and an enrichment of CD68^+^ TAMs in GBM-1 and GBM-2 (Figure 4A). Consistent with the immunostaining, immune deconvolution of the CD45^+^ immune samples revealed an enrichment of microglia and macrophages in GBM-1 and 2, while in GBM-3, a substantial increase in lymphocytes and neutrophils was identified (Figure 4B). Investigating each sample individually revealed an increased lymphocyte population in regions with a high proportion of mesenchymal cells (correlation: 0.58) and the astrocytic lineage correlated with TAM cell types (correlation: 0.72, Supplementary Figure 3A). Enrichment in BDMs is classically associated with the expression of macrophage gene *AHR*, while microglia are associated with *P2RY12* and *TMEM119* (Supplementary Figure 3B), however, was further refined using the 859 gene signature (Supplementary Figure 3A). Deeper deconvolution analysis of each sample identified contamination with neoplastic cells, likely during the capture process (Supplementary Figure 3C). Immune cell proportions were unchanged independent of the addition of neoplastic cell types to the deconvolution reference (Supplementary Figure 3D), validating our use of the deconvolution method. To spatially discern the patterns of immune cell infiltration, we mapped the samples back onto the sample H&E (Figure 4C). Regions located close to one another on the same tumor piece displayed vastly different immune profiles, consistent with the distribution of tumor cell states, together suggesting a strong link between tumor cell and immune infiltrate in the local microenvironment.

To dissect the immune subtypes and location at a single-cell level, the immune cluster in the single-cell data of GBM-1 was extracted and reclustered (Supplementary Figure 4A). Gene expression analysis revealed a cluster with a dominant expression of *AHR*, denoting the identification of BDMs (Supplementary Figure 4B), opposed to microglia. The proportions of immune cells in GBM-1 from the GeoMx immune samples and single-cell CosMx analysis revealed similar predictions from each technology, validating both our analysis approaches (Supplementary Figure 4C). Single-cell analysis of the immune population revealed tumor and immune cells revealed dispersion of immune cells throughout the field of view, with a heightened concentration of immune cells surrounding vasculature (Figure 4C i, ii, Supplementary Figure 5). To investigate the direct interactions that may be driving immune cells to co-occur more frequently within different tumor niches, we performed ligand/receptor analysis on the whole transcriptomics data from GBM samples investigated with GeoMx (Figure 4D). Analysis of a cohort of tumor to immune genes revealed patterns of communication, such as signaling via the TGFβ pathway, where samples enriched for *TGFBR1*, *TGFBR2* expression saw significantly increased BDM and microglia infiltrate (correlation: 0.88, *P* value <.001), associated with increased *TGFB1, TGFB2* ligand expression from tumor cells. Conversely, increased *ACVR1B, TNFRSF13B* receptor expression correlated with neutrophil and lymphoid infiltrate (correlation: 0.58), associated with *TDGF1, TNSF13* tumor ligand expression. Together, these data suggest subtype-specific interactions between tumor and immune infiltrate in the tumor microenvironment.

### Glioblastoma architecture reveals distinct cell state niches

**Figure 5.**
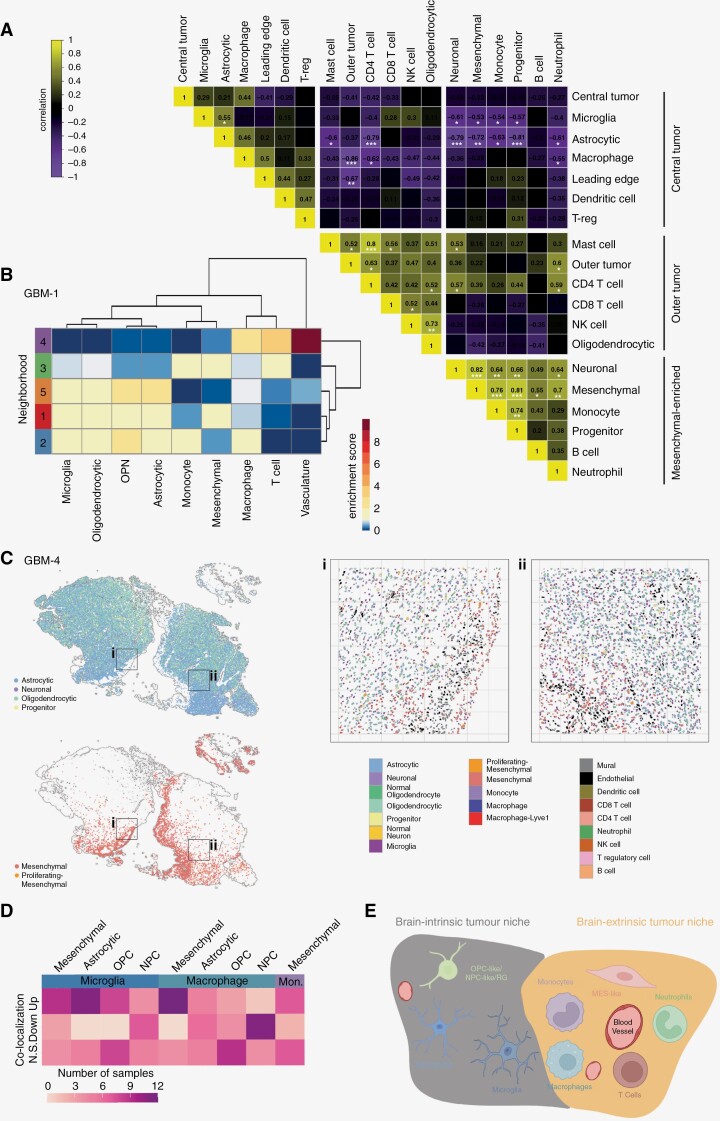
Tumor and immune cell types associate in discrete neighborhoods. (A) Correlation plot of immune cell types, tumor cell states, and location classifications across matched GBM immune samples (*n* = 15). Correlation value and *P* value inset in each square. (B) Enrichment heat map of each cell type identified co-localized within the neighborhoods in GBM-1. (C) Location of glial-derived tumor cell states (astrocytic, neuronal, oligodendrocytic, and progenitor; top) compared to mesenchymal cell states (bottom) in GBM-4. Zoom-in Voronoi plots of (i) and (ii) displaying all cell types. (D) Number of Visium samples^15^ with significant positive or negative co-localization between tumor and immune cell types. (E) Schematic of tumor and immune architecture in GBM samples highlighting key findings from this study.

To investigate these associations, we next interrogated co-localization of our deconvoluted cell states in addition to physical locations in the tumor. Correlation between the cell states identified 3 domains: central tumor, outer tumor, and mesenchymal enriched. Within the central tumor domain, astrocytic cells and microglia associate with the tumor, while the tumor edge is associated with dendritic cells (Figure 5A). Consistent with poor infiltration of lymphocytes, T cells, mast cells, and NK cells were enriched in the outer tumor domain, while the mesenchymal tumor cells defined a distinct domain rich in monocytes, neutrophils, B cells, and neural progenitor cells. We observed a strong relationship between mesenchymal cells and monocytes (correlation: 0.76). To investigate the spatial association of tumor and immune cells within their microenvironment, we investigated the distribution using the single-cell maps in GBM-1. We found that the mesenchymal tumor cells were found more frequently in clusters, as were monocytes and macrophages (Supplementary Figure 6A). To identify which cells were found more frequently together, we performed a neighborhood analysis, defining 5 unique niches, characterized by distinct groups of cells, likely dictated by secreted factors in the microenvironment and receptor/ligand interactions (Supplementary Figure 6B). The most distinct cellular architecture is observed in neighborhood 4, which is dominated by vasculature (endothelial and mural cells) together with T cells and macrophages, captured in the perivascular space (Figure 5B, Table S3). Consistent with the correlation analysis, mesenchymal cells and monocytes formed neighborhood 3 and were frequently within the same microenvironment. Neighborhood 5 revealed a brain-intrinsic tumor niche (astrocytic, oligodendrocytic, and progenitor cell states) together with the microglial cells. This association represents a classical feature of the brain microenvironment under normal conditions, where resident microglial cells associate with glial cells in the absence of lymphocyte infiltration.^14,23,24^ In contrast, the mesenchymal tumor cells are more broadly associated with circulating immune cells, including macrophages, T cells, and monocytes in neighborhoods 1–3 (Figure 5B).

To further investigate tumor cell domains in an independent single-cell cohort, we used the GBM-4 Xenium dataset. Consistent with the findings in GBM-1 neighborhoods, we identified a clear brain-intrinsic tumor niche, where astrocytic, oligodendrocytic, progenitor, and neuronal cells co-located, while mesenchymal tumor cells were in distinct regions (Figure 5C). Due to the larger area analyzed in this dataset, we achieved greater cellular resolution and could interrogate all tumor and immune populations (Supplementary Figure 7A, B) and performed an in-depth neighborhood analysis. Neighborhoods 3 and 6 were rich in mesenchymal cells and associated with macrophages, monocytes, and other immune populations, while neighborhoods 1, 5, and 8 were rich in brain-intrinsic tumor cells (Supplementary Figure 7C, D). Due to the higher number of cells analyzed, we observed co-localization within populations for not only mesenchymal cells but also astrocytic and oligodendrocytic tumor cell states, consistent with the increased cell density of GBM-4 (Supplementary Figure 7E). Together, these findings extend upon those of GBM-1.

To interrogate this observation to a larger independent dataset, we investigated spatial transcriptomic data from an independent cohort of 16 GBM tumors analyzed by Visium technology.^14^ Using deconvolution of cell types in each spot, we performed co-localization analysis (Figure 5D). We found that microglia most frequently associated with astrocytic tumor cells and macrophages and monocytes strongly associated with mesenchymal tumor cells. Investigating the relationship between monocytes and mesenchymal tumor cells in a representative GBM sample revealed distinct patches of mesenchymal regions with monocyte infiltration absent from regions rich in brain-intrinsic tumor cell types (Supplementary Figure 7F). Taken together, we identify consistent patterns of tumor architecture and immune infiltration across two cohorts of GBM using three independent spatial transcriptomic technologies (Figure 5E, Supplementary Figure 8). We find a brain-intrinsic tumor niche comprised of astrocytic and oligodendrocytic tumor cell states associated with the brain resident microglia. A second niche we call the brain-extrinsic tumor niche contains mesenchymal patches infiltrated by monocytes. Within these patches, neutrophils, T cells, monocytes, and macrophages are located near blood vessels. Collectively, our results provide a model of the GBM microenvironment encompassing discrete niches characterized by the activity of separate signaling pathways.

## Discussion

Here, we provide a spatial characterization of the tumor microenvironment in *IDH* wild-type and *IDH1*-mutant HGG, revealing vast heterogeneity and discrete interactions between tumor cell states and immune cells within a highly organized tumor architecture. Although past studies have identified tumor cell states in glioma and shown the vast heterogeneity of these cell states within individual tumors,^13,32,33^ this is the first study to spatially interrogate transcriptional heterogeneity of glioma at a single-cell level. Through the adaptation of bioinformatic tools to analyze new datasets of spatial transcriptomics (digital spatial profiling and single molecule imaging), we observe immense molecular and cell composition heterogeneity, which was conserved across both independent cohorts of *IDH-*wt GBM samples and spatial transcriptomics technologies.

Recent stratification of single cells in GBM based on cell lineages of the normal fetal brain characterized five cell states in two broad groups.^13^ The normal neural lineages: astrocytic, oligodendrocytic, and neuronal, together with the proliferative progenitor cell state; and the mesenchymal lineage which does not have a direct parallel in the normal brain. Characterization of samples and regions into these five cell states via deconvolution yields different results compared to previous bulk classifiers.^20,31^ This is likely attributed to the significant increase in the number of markers used^13^ and the isolation of the tumor and immune compartment. Stratification by deconvolution opposed to bulk classifiers, particularly on samples with isolated tumor cells, offers more granularity and is thus of greater utility in a spatial context. Similar to previous studies that rely on multisite sampling from different anatomical locations,^34^ we demonstrate tumors contain multiple subtypes sometimes in close proximity. We extend these initial observations in the context of their spatial relationship with the immune compartment, which defines a brain-intrinsic niche—comprised of astrocytic, oligodendrocytic, neuronal, and progenitor cell states—closely associated with the resident microglial cells and rarely interacting with immune cells migrated from the circulation. In contrast, a brain-extrinsic niche characterized by mesenchymal tumor cells, closely associated with macrophages, monocytes, and other lymphocytes, is consistent with recently described mesenchymal patches in GBM that are clearly spatially demarcated,^14^ and thought to be linked to tumor progression.^35^ The nature of immune infiltration in mesenchymal patches concurs with work by Varn et al. finding that myeloid cells in mesenchymal tumor subtypes are likely to be macrophages.^20^ More recently, work by Albiach et al.^36^ suggests that these mesenchymal patches are domains characterized by wound healing and hypoxia response in glial cells with infiltration by monocytes and macrophages, displaying reproducible organization consistent with our findings. We also show that the conditions in brain-intrinsic and -extrinsic tumor niches are likely dictated by specialized ligand/receptor interactions in agreement with Gangoso et al. who demonstrated tumor state-specific transcriptional and epigenetic changes reshape the immune microenvironment.^21^ The immune and regulatory underpinning of the dichotomy of the glioma microenvironment suggests that the success of immunotherapy agents such as chimeric antigen receptor T-cell infusion, checkpoint inhibitor blockade, or dendritic cell therapies,^37^ may be influenced by tumor composition.

In *IDH*-mutant glioma, scRNAseq analysis identified the presence of cell states restricted to the astrocytic and oligodendrocytic lineages.^16,17^ Here, we demonstrate the dominance of the oligodendrocytic lineage across both astrocytoma and oligodendroglioma samples, conserved spatially throughout the tissue. The neuronal lineage was also present in these tumors and increased in frequency as the tumor purity declined, likely representative of normal neural cells. Notably, progenitor cells were identified in the tumor regions, and stratified to the proliferative tumor compartment only, consistent with previous reports of a rare subpopulation of progenitor cells.^16^ Indeed, the intratumoral heterogeneity of *IDH1-*mutant HGG was substantially reduced in comparison to *IDH-*wt GBM. This marked difference in heterogeneity between grade 3 and grade 4 gliomas has been postulated to reflect a more lineage-restricted cell-of-origin in *IDH-*mutated tumors.^17^

Heterogeneity is a hallmark of GBM and has been on display in this spatial analysis in comparison to astrocytoma, as in other recent GBM datasets,^14,15^ with implications for the limitations of personalized therapies for patients. Further, spatial heterogeneity highlights the challenge of identifying biomarkers for treatment from potentially un-representative diagnostic sampling, and will likely create a hurdle in future clinical decision-making. We, and others, have highlighted the association of the mesenchymal lineage with increased infiltration of circulating immune cells and as described above, may represent an avenue to leverage immunotherapeutic approaches, in patients who are more likely to respond. This is in contrast to chemotherapy treatment, whereby mesenchymal cells appear to be resistant to standard therapy and are frequently observed in recurrent tumors,^19,35^ requiring novel treatment strategies. Thus, routine mechanisms to predict the proportion of mesenchymal lineage cells would likely aid in diagnostic and prognostic predictions. Future studies employing high-plex spatial molecular imaging with an increased sample size will aid in the further characterization of spatially distinct domains to identify mechanisms of plasticity and immune infiltration and their secreted factors in patients with HGG. Ultimately, understanding the spatial distribution of tumor cells within their microenvironment will provide clinical impact with the potential to selectively modulate cellular plasticity and reshape the landscape of therapeutic targeting in GBM.

## Supplementary Material

vdad142_suppl_Supplementary_Material

## Data Availability

The GeoMx^®^, CosMx^®^ and Xenium^®^ data is available in public repositories located in the methods section. All code required for the analysis of the data can be found at: https://github.com/SaskiaFreytag/spatial_brain_cancer/.
