# Supplementary material for: Spatial architecture of high-grade glioma reveals tumor heterogeneity within distinct domains
Source: Neurooncol Adv. 2023 Nov 1;5(1):vdad142. doi: 10.1093/noajnl/vdad142 (PMC10699851; doi:10.1093/noajnl/vdad142)
Supplement: vdad142_suppl_Supplementary_Material [file vdad142_suppl_supplementary_material.pdf]

Supplementary Materials for

Spatial architecture of high-grade glioma reveals tumor heterogeneity within distinct domains

Joel Moffet *et al.*

Corresponding authors: Sarah A. Best, E-mail: best@wehi.edu.au; Saskia Freytag, E-mail: freytag.s@wehi.edu.au; James R. Whittle, E-mail: whittle.j@wehi.edu.au;

This PDF file includes:

Methods Supplement

Supplementary References

Supplementary Figures 1 to 7

Supplementary Tables 1 to 5

Methods Supplement

Pathology Review

Pathology review was performed on the six cases. The tissue sections demonstrated distinct morphological features and were assessed using the cellularity of the tumor cells, nuclear pleomorphisms, presence of mitoses, necrosis, and microvascular proliferation. The five anatomic features proposed by The Ivy Glioblastoma Atlas Project (Ivy GAP) were used to annotate the tumors into regions. These included: Leading edge (LE): the outermost part of tumor with the least tumor cell density and bordering on the normal tissue area; Cellular tumor (CT): the core of the tumor with highest cell density; Infiltrating tumor (IT): the zone interposed between LE and CT with moderate tumor cell density; Microvascular proliferation (MVP): Areas with endothelial proliferation; Pseudopalisading cells around necrosis (PAN): dense tumor cells geographically fenced around core necrosis. Other features recognized are regions of large non-palisaded necrosis (NEC) and normal brain tissue (N). Annotations were performed by two pathologists.

The evaluation of Ki67 and CD45 immunostaining was performed according to standard established protocols using light microscopy. The percentages of tumor Ki67 positive cells were assessed semi-quantitatively. All positive cells were counted irrespective of the staining intensity. 200 tumor cells were counted in hotspot areas and the percentage was calculated by dividing the number of positive cells by the total number of tumor cells in the tumor areas assessed. CD45 immunostaining (**Table 1**) was evaluated using a categorical scoring system[1–3]. All positive cells were counted irrespective of the staining intensity. Whole tissue sections were scanned, and the density was semi-quantitatively assessed as either nil, sparse, moderate or dense. CD45, along with CD3 and CD68 (as shown in Table S3), underwent additional objective quantification, and the results were presented as percentages. These quantifications were then analyzed with respect to their respective topographical locations within the tumor, including the tumor parenchyma, perivascular space, and areas of necrosis. Two representative fields per location within each tumor were selected. The sections were exported to Qupath software for automated cell quantification using a custom pixel classifier trained to quantify the percentage of positive cells. To establish ground truth, whole slide images containing both positively and negatively stained cells, representing stained and unstained cells, respectively, were utilized.

GeoMx® Dataset

Experimental procedure

After baking the slides for 2 hours for paraffin removal, slides were loaded onto a Leica BOND RX for tissue rehydration, heat-induced epitope retrieval (ER2 for 20 minutes at 100 °C) and proteinase K treatment (0.1 µg/ml for 15 minutes at 37 °C). The tissue sections were then hybridized with the Human Whole Transcriptome Atlas (WTA) probes overnight. Following 2 x 5 min stringent washes (1:1 4x SSC buffer & formamide), the slides were blocked and then incubated morphology marker antibodies: GFAP (488 channel, NBP2-3318AF488, Novus), CD45 (594 channel, 13917BF, Cell Signaling Technologies), and Ki67 (647 channel, 9027BF, Cell Signaling Technologies). Syto83 (532 channel, S11364, Invitrogen) was used as a nuclear stain. Tissue sections were then loaded into the GeoMx® Digital Spatial Profiling (DSP) platform, which is a histology-based platform where oligo-barcodes are attached via a photocleavable linker to fluorescent RNA *in situ* hybridization probes. Paired with histology staining individual populations in the intermixed regions were selected (non-cycling tumor: GFAP/ DAPI; cycling tumor: Ki67/GFAP/DAPI; immune: CD45/DAPI; **Tables S1 and S2**). UV light was then directed by the GeoMx® at each sample and released the RNA ID and UMI-containing oligonucleotide tags from the WTA probes for collection and sequencing preparation. Illumina i5 and i7 dual indexing primers were added to the oligonucleotide tags during PCR to uniquely index each sample. AMPure XP beads (Beckman Coulter) were used for PCR purification. Library concentration as measured using a Qubit fluorometer (Thermo Fisher Scientific) and quality was assessed using a Bioanalyzer (Agilent). Sequencing was performed on an Illumina NovaSeq 6000 and .fastq files were processed into gene count data for each sample using the GeoMx® NGS Pipeline. Read depth was performed with 79,915,933 total transcripts identified across 75,951 nuclei, corresponding to an average of 1,052 transcripts per nucleus.

Preprocessing

For all computational analysis methods were run using defaults unless otherwise specified. Raw counts from the two separate experimental batches were grouped into a single dataset. Samples were removed if their nuclei count was below 100 nuclei, as provided by NanoString. Genes were filtered out unless above limit of quantitation in 5 % of remaining samples, calculated for each

sample as the geometric mean of all negative probes multiplied by the geometric standard deviations of the negative probes to the second power.

From filtered raw counts of each region, a combined dataset was constructed, where Ki67 (K) and $Ki67^-$ (T) tumor samples from the same region were joined together into a single tumor sample ([T + K]). This was done to ensure the entire neoplastic compartment of each region was being compared when desired. Further preprocessing of both the original separated [T] + [K] dataset and the combined [T + K] dataset were performed independently but identically as follows.

Remaining genes were normalized by upper quartile normalization. Batch correction was required as we observed different signal-to-noise ratios, as previously observed[4]. Here we performed batch correction on the logged normalized count data, using limma via geomxBatchCorrection in standR (v1.0.0), separately for astrocytoma and GBM, with experimental batch and sex considered. Correcting for batch effects on all astrocytoma and GBM samples simultaneously showed inappropriate mixing of tumor classes in PCA and suggested loss of biologically relevant differences. PCAs were plotted using functions from the standR package (v1.0.0). Cell cycle scoring was estimated by Seurat (v4.1.1). Hypoxia levels were measured by abundance of genes in each sample from the respective KEGG pathway using the keggGet function (KEGGREST v1.36.3).

Deconvolution

The tumor deconvolution reference was constructed from Couturier *et al.* dataset with unassigned tumor cells excluded[5]. The top 5,000 highly variable genes also present in the GeoMx® filtered gene list were used to form signature profiles for each cell state, via the spatialDecon package (v1.6.0). Testing for difference in cell state proportions between astrocytoma and GBM as well as $Ki67^+$ and $Ki67^-$ regions was performed using propeller in the speckle package (v0.0.3), using t-tests and estimated number of nuclei of each cell state. A logit transformation was used to test the proportions and multiple testing was corrected using false discovery rate (FDR).

The immune deconvolution reference was constructed from Ruiz-Moreno *et al.* dataset[6] via the spatialDecon package (v1.6.0). Immune cell type signatures were derived from Ruiz-Moreno 'cell

type' annotation; for segregating T cells, only cells that were labelled as 'mature T cell' in the 'cell_type' annotations and contained either 'CD4', 'CD8', or 'reg' in the 'celltype_original' annotations were included for each respective cell type. Alongside the immune cell types, a neoplastic signature and non-immune non-neoplastic signature were also included from 'annotation_level_1,' to account for possible contamination of immune samples. All genes intersecting the safeTME profile as well as the GeoMx® filtered gene list were included (n = 859). Testing for differences in cell type proportions between classifications was performed using propeller in the speckle package (v0.0.3), using F-tests via ANOVA and estimated number of nuclei of each cell state. A logit transformation was used to test the proportions and FDR was applied.

Classifications

Verhaak classifications employed single sample Gene Set Enrichement Analysis (ssGSEA) of the top 50 genes for each gene set signature, as previously performed by *Varn*[7]. *Garofano* subtype assignment used the online app (https://lucgar88.shinyapps.io/GBMclassifier) developed to infer likeliest subtype from lognormalized count data[8]. As both classifications are based upon the combined tumor microenvironment, CD45, Ki67^{+} and Tumor compartments from the same region were combined into one expression profile.

DEG

DEG analysis was performed with limma-voom (limma v3.52.4) /edgeR (v3.38.4). Voom was applied twice, with duplicate correlation considered both times for samples coming from the same region. Sex and Batch were also added to the model where appropriate. GO term analysis was performed on DEG lists using goana, with trend set to TRUE (limma v3.52.4). GO terms were then clustered using semantic clustering via simplifyEnrichment (v1.6.1), and further clustered with kappa clustering to separate into groups of similarly worded GO terms that share similar gene sets.

Shannon Entropy

Shannon entropy across all individual genes was calculated from upper-quartile normalized counts using a shrinkage estimator to counter the effect of under sampling for lower nuclei samples.

Correlation plot

GBMs with matched immune and tumor samples were correlated along with their location classifications based on immunostaining, employing the combined [T + K] dataset. Regions labelled as ‘Ki67 Border’ and ‘Normal regions’ constituted the ‘Outside’ locations, and regions labelled as ‘Border’ formed the ‘Edge’ location signature. Significance was assessed by linear regression, using the Pearson product-moment correlation coefficient as the test statistic. The correlation plot and significance tests were processed using corrplot (v0.92).

Ligand-receptor analysis

Ligand-receptor analysis employed the connectomeDB2020 list of literature-supported ligand-receptor pairs. Only regions with matching tumor and immune samples were included. Correlations between ligand and receptor expression was calculated in the same sample (immune to immune, and tumor to tumor signaling) and in matched sample pairs (tumor to immune, and immune to tumor signaling). Displayed ligand-receptor pairs were manually selected based on the recurrent increased correlation of particular signaling pathways in the same direction (for example, increased correlation of WNT signaling pairs in tumor to tumor interactions). Spiderplots were constructed with ggradar (v0.2).

CosMx® Dataset

Experimental procedure

Tissue samples were obtained and prepared at 5 µm for analysis as previously described[9]. Serial sections were H&E stained to identify regions for RNA target readout and protein detection. We then selected 12 FOVs of 0.9 x 0.7 mm size. RNA readout was performed by flowing 100 µl of Reporter Pool 1 (SMI 1,000 plex RNA panel, **Table S4**) into the flow cell and incubating for 15 min. Reporter Wash Buffer (1 mL) was then flowed to wash unbound reporter probes, and Imaging Buffer was added for imaging. Nine Z-stack images (0.8 µm step size) for each region were acquired, and photocleavable linkers on the fluorophores of the reporter probes were released by UV illumination and washed with Strip Wash buffer. The fluidic and imaging procedure was repeated for the 16 reporter pools, and the 16 rounds of reporter hybridization-imaging were repeated multiple times to increase RNA detection sensitivity, as is routinely performed by NanoString[9]. After RNA readout, the tissue samples were incubated with a 4-fluorophore-

conjugated antibody cocktail against Histone H3, 18s rRNA, GFAP and DAPI stain in the CosMx® instrument for 2 h. Imaging Buffer was added to the flow cell and nine Z-stack images for the 4 channels (3 antibodies and DAPI) were captured. We were only able to analyze 7 FOVs of the 12, as 5 FOVs were not in adequate locations for analysis and of poor quality with low numbers of cells and transcripts detected.

Cell segmentation

For cell segmentation the NanoString pipeline combining image preprocessing and machine learning techniques was used on tissue images stained with Histone H3, 18s rRNA, GFAP and DAPI. Briefly, the pipeline first performs pre-processing for boundary enhancement followed by cell segmentation via a pretrained neural network in Cellpose (v2.0).

Preprocessing

We next generated single-cell expression matrix by counting molecules of each gene within the area assigned to a cell by the segmentation algorithm. We removed any cells with fewer than 20 total transcripts or more than 3 negative control probes. Cells were also removed if they had fewer than <20 genes detected. After removal, the average cell had 80.9 detected number of unique genes and 174.4 number of transcripts. Furthermore, the gene panel was well utilized with 574 genes being detected in at least 5% of cells. We then applied a log-normalization to the expression matrix using scater (v1.26.1) in combination with SpatialExperiment (v1.9.4). Highly expressed *MALAT1* was removed before any subsequent analyses.

Cell type annotation

Data dimensions were reduced using PCA and the number of components kept was determined via a global maximum likelihood based on translated Poisson mixture model approach with 20 nearest neighbors implemented in intrinsicDimension (v1.2.0). This approach has recently been shown to produce the best separation of challenging subpopulations. We also generated a Uniform Manifold Approximation and Projection (UMAP) embedding based on the PCA with *spread* set to 3 and *mindist* set to 0.01 with 15 nearest neighbors. Cells were then clustered based on the PCA via Leiden clustering implemented in bluster (v1.8.0). Cells were automatically annotated using

SingleR (v2.0.0) with the reference set as the downsampled GBM harmonized dataset[6] (see description in Deconvolution).

Clustering of the entire dataset produced 35 clusters. Clusters were first manually annotated as Vasculature, Immune or Tumor according to the predicted cell type labels from SingleR and marker genes (Vasculature: *COL4A2*, Tumor: *APO-J*, Immune: *CD163*). Cells in the immune and tumor compartment were reclustered following the process outlined above, including dimension reductions (during the immune cell reclustering we identified further mesenchymal cells). This produced 16 clusters for the immune cells and 27 clusters for the tumor cells. Cell type labels for the tumor cells were additionally predicted using SingleR with the reference set to the Couturier *et al.* dataset[5]. Clusters for both immune and tumor cells were annotated according to the predicted labels and known markers genes. Note that 96% of tumor cells contained at least one detected marker gene.

Networks and modularity

Using reticulate (v1.28), the preprocessed dataset was converted to an *anndata* object to be analyzed by squid.py (v1.2.3). We were then able to build an adjacency matrix indicating the cells that are in ~30µm radius (179 pixels) of each cell within the same region. By combining the adjacency matrix with the cell type annotations for each cell the modularity of each cell type can be worked out using igraph (v1.4.0). The modularity measures how strongly separated cells of the same type are compared to a random null model with higher values showing less spatial separation between cells of the same cell type.

Neighborhood clustering

To identify tumor niches, we developed our own version of a neighborhood composition approach. Briefly, we first divide each region into overlapping 500 by 500 pixel windows that are shifted by 100 pixels. On average each window contained 107.5 cells (median 51 cells). In each window, we count the number of different cell types. Using a Bray-Curtis dissimilarity implemented in vegan (v2.6-4), we built a hierarchical clustering tree utilizing *hclust.* With the help of the hierarchical clustering tree, we decided on 5 clusters and identified cluster labels for each window using *cutree*. As each 100 by 100 pixel tile of each region is overlapped by multiple windows with an associated

cluster label, we use a majority voting strategy to identify the cluster label for each 100 by 100 pixel tile. This results in neighborhoods being identified across the regions, which can be described by their enrichment of cell types using a simple chi-square test approach.

Xenium® Dataset

Gene panel design

The Xenium® In Situ technology uses targeted panels to detect gene expression. 339 genes for cell type identification were selected and curated primarily based on single cell atlas data of GBM (**Table S5**). Probes contain two complementary sequences that hybridize to the target RNA and a third region encoding a gene-specific barcode. This design ensures high specificity by preventing off-target signals. Upon probe binding to the target RNA, the paired ends ligate to generate a circular DNA probe.

Experimental procedure

We first sectioned a 10 µm FFPE tissue section onto a Xenium® slide (12 x 24 mm), followed by deparaffinization and permeabilization to make the mRNA accessible. Probe hybridization of genes and 2 negative control genes occurred at 50 °C overnight with a probe concentration of 10 nM. This was followed by stringency washing to remove un-hybridized probes and another round of probe ligation and annealing of rolling circle amplification primer at 37 °C for two hours. The resulting circularized probes were then enzymatically amplified for one hour at 4 °C followed by two hours at 37 °C. After washing, background fluorescence was quenched chemically.

Sections were then imaged using the fully automated Xenium® Analyzer instrument. On the Xenium® Analyzer, image acquisition is performed in 15 cycles that include reagent cycling, incubation, fluorescent probe hybridization, imaging, and probe removal. In each cycle, fluorescently-labeled oligonucleotides are bound to amplified barcodes and the fluorescent intensity in each of the four Xenium® color channels is measured, which is used to identify a target gene. Only transcripts that had a Quality Value >20 were kept. Z-stacks were taken with a 0.75 µm step size across the entire tissue thickness, which are stitched back together to make a spatial map of the transcripts across the tissue.

Nuclei segmentation

To assign mRNAs to nuclei, and thus enable downstream analysis, DAPI images were used to detect nuclei using 10x Genomics supplied neural network algorithm.

Preprocessing

We next generated a single-cell expression matrix by counting molecules of each gene within the nucleus-bound area assigned to a cell by the segmentation algorithm. We removed any cells with fewer than 20 total transcripts within the nucleus. We then applied a log-normalization to the expression matrix using scater (v1.26.1) in combination with SpatialExperiment (v1.9.4).

Cell type annotation

Data dimensions were reduced using PCA and the number of components kept was determined via a global maximum likelihood based on translated Poisson mixture model approach with 20 nearest neighbors implemented in intrinsicDimension (v1.2.0). This approach has recently been shown to produce the best separation of challenging subpopulations. We also generated a Uniform Manifold Approximation and Projection (UMAP) embedding based on the PCA with *spread* set to 3 and *mindist* set to 0.01 with 15 nearest neighbors. Cells were then clustered based on the PCA via Leiden clustering implemented in bluster (v1.8.0). Cells were automatically annotated using SingleR (v2.0.0) with the reference set as the downsampled GBM harmonized dataset[6] (see description in Deconvolution).

Clustering of the entire dataset produced 114 clusters. Clusters were first manually annotated as Vasculature, Immune, Neuron, Oligodendrocyte or Tumor according to the predicted cell type labels from SingleR and marker genes (Vasculature: IGFBP4, Tumor: PTPRZ1, Immune: PTPRC, Neuron: C1QL3, Oligodendrocyte: ERMN). Cells in the immune and tumor compartment were reclustered following the process outlined above, including dimension reductions (neurons were included within the tumor reclustering). This produced 76 clusters for the immune cells and 150 clusters for the tumor cells. Cell type labels for the tumor cells were additionally predicted using SingleR with the reference set to the Couturier *et al.* dataset[5]. Clusters for both immune and tumor cells were annotated according to the predicted labels and known markers genes. From the immune compartment, lymphoid cell types were further reclustered as above to delineate T cell populations

and NK / B cells. This produced 8 clusters that were annotated according to the predicted labels and known markers genes.

Networks and modularity

Mimicking the analysis performed on the CosMx® dataset, the preprocessed dataset was converted to an *anndata* object to be analyzed by squid.py (v1.2.3). We were then able to build an adjacency matrix indicating the cells that are in 30 µm radius of each cell within the same region. By combining the adjacency matrix with the cell type annotations for each cell the modularity of each cell type can be worked out using igraph (v1.4.0).

Neighborhood clustering

To identify tumor niches, we adjusted the neighborhood composition approach used for the CosMx® dataset. Due to the larger sample area increasing the computational intensity of the analysis, window size was expanded to 160 µm by 160 µm, with each window shifted by 40 µm. With the help of the hierarchical clustering tree, we decided on 8 clusters and identified cluster labels for each window using *cutree*.

Visium® Dataset

Processed data were downloaded from https://doi.org/10.5061/dryad.h70rxwdmj. Deconvolution of spots as described in Ravi *et al.* were obtained from the authors upon request[10].

Co-location testing

To test co-location of two cell types, we modified a method for the identification of proximal interacting cell types in Giotto. We first determined whether the cell types were present in any spot by multiplying the predicted proportion with the number of cells in the spot. Like for the SMI data, we built an adjacency matrix indicating the direct neighbors for each spot. Using this adjacency matrix, we can then find the number of times the cell types of interest are co-located. By permuting the cell type labels 1000 times, we can find distribution of the number of expected co-location events, which allows us to determine a z-score and can be tested. P-values from tests were corrected for multiple testing using *p.adjust* with a FDR method.

Supplementary References

1. Dahlin, A. M. *et al.* Colorectal cancer prognosis depends on T-cell infiltration and molecular characteristics of the tumor. *Mod. Pathol.* **24**, 671–682 (2011).
2. Samman, D. M. E. *et al.* Immunohistochemical expression of programmed death-ligand 1 and CD8 in glioblastomas. *J. Pathol. Transl. Med.* **55**, 388–397 (2021).
3. Maddison, K. *et al.* Low tumour-infiltrating lymphocyte density in primary and recurrent glioblastoma. *Oncotarget* **12**, 2177–2187 (2021).
4. van Hijfte, L. *et al.* Alternative normalization and analysis pipeline to address systematic bias in NanoString GeoMx Digital Spatial Profiling data. *iScience* **26**, 105760 (2023).
5. Couturier, C. P. *et al.* Single-cell RNA-seq reveals that glioblastoma recapitulates a normal neurodevelopmental hierarchy. *Nat. Commun.* **11**, 1–19 (2020).
6. Ruiz-Moreno, C. *et al.* Harmonized single-cell landscape, intercellular crosstalk and tumor architecture of glioblastoma. *bioRxiv* 2022.08.27.505439 (2022) doi:10.1101/2022.08.27.505439.
7. Varn, F. S. *et al.* Glioma progression is shaped by genetic evolution and microenvironment interactions. *Cell* **185**, 2184-2199.e16 (2022).
8. Garofano, L. *et al.* Pathway-based classification of glioblastoma uncovers a mitochondrial subtype with therapeutic vulnerabilities. *Nat Cancer* **2**, 141–156 (2021).
9. He, S. *et al.* High-plex imaging of RNA and proteins at subcellular resolution in fixed tissue by spatial molecular imaging. *Nat. Biotechnol.* (2022) doi:10.1038/s41587-022-01483-z.
10. Ravi, V. M. *et al.* Spatially resolved multi-omics deciphers bidirectional tumor-host interdependence in glioblastoma. *Cancer Cell* **40**, 639-655.e13 (2022).

Supplementary figure 1

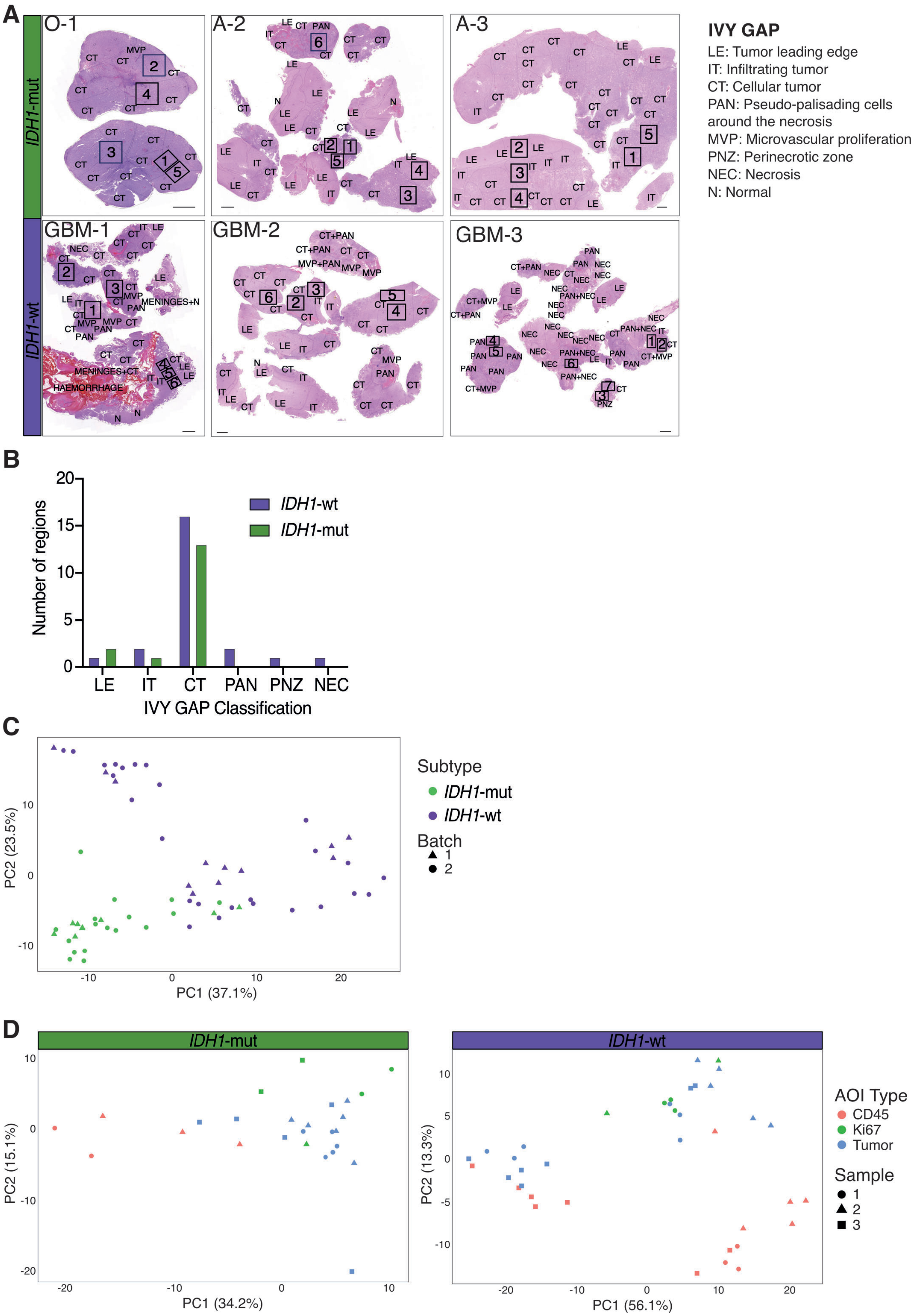

Supplementary Figure 1 |

A. H&E of samples with pathology annotation and regions of analysis indicated. Scale, 1 mm. **B.** Number of regions selected relative to Ivy GAP pathology annotation. **C.** Principal component analysis of all samples. Displaying first two principal components for all samples following batch correction, colored by tumor type. **D.** Principal component analysis of *IDH1*-mut (left) and *IDH1*-wt (right) samples. Displaying first 2 principal components following batch correction, colored by sample type.

Supplementary figure 2

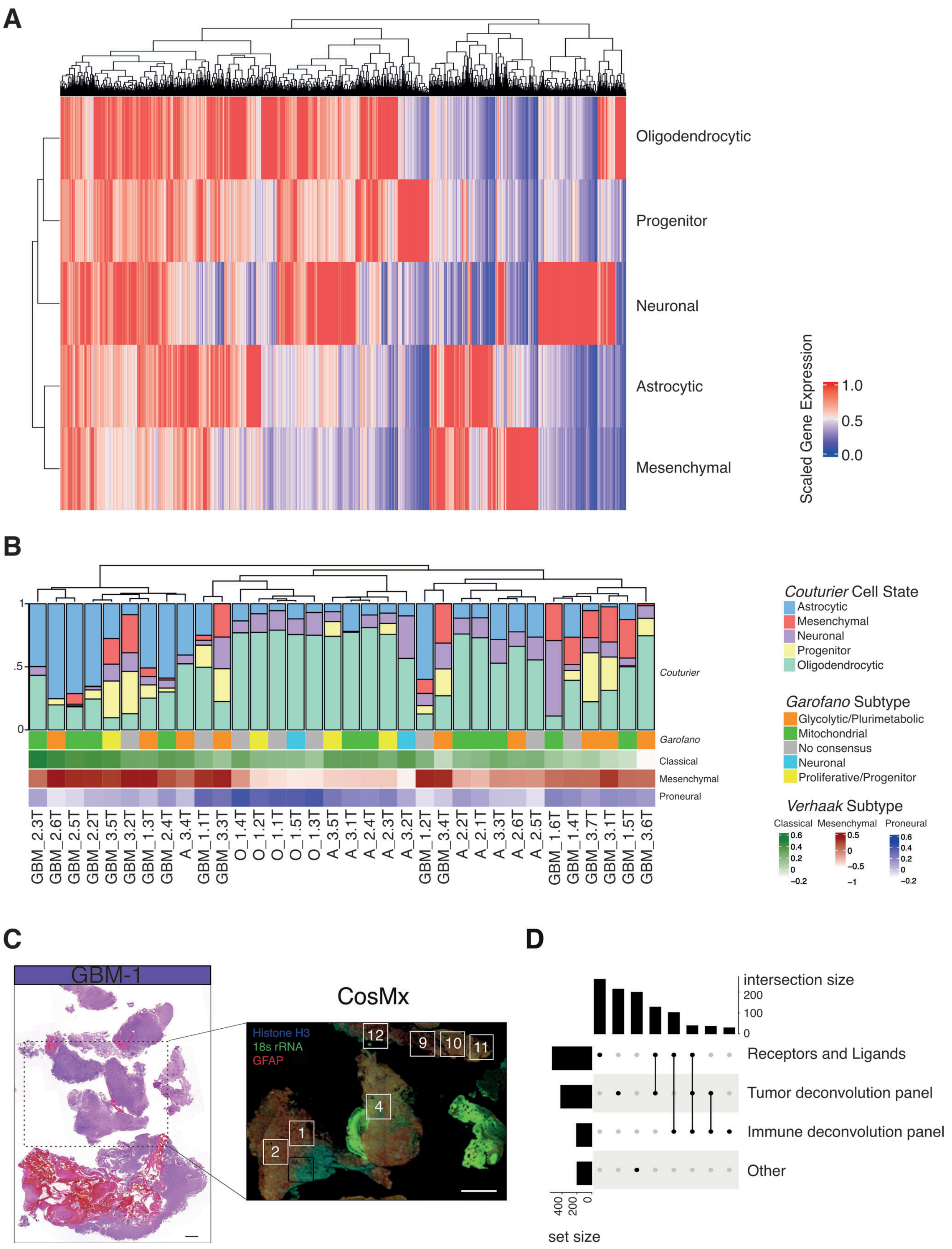

Supplementary Figure 2 |

A. Deconvolution heatmap signature for tumor cell states. Scaled gene expression of top 5,000 highly variables genes from *Couturier*[5] dataset present in preprocessed gene list, for each cell state profile. **B.** Analysis of tumor cell states using *Couturier*[5] cell states (astrocytic, mesenchymal, neuronal, progenitor, oligodendrocytic), *Garofano*[8] subtypes (glycolytic/plurimetabolic, mitochondrial, neuronal, proliferative/progenitor) and *Verhaak*[7] subtypes (classical, mesenchymal, proneural). **C.** Placement of single cell spatial analysis regions relative to GeoMx® regions. Scale, 1 mm. **D.** UpsetR plot of NanoString 1,000 onco-immunology panel. The number of probes represented in single signatures and the number of overlapping probes are visualized to generate the tumor and immune cell state signatures and for ligand/receptor analyses.

Supplementary figure 3

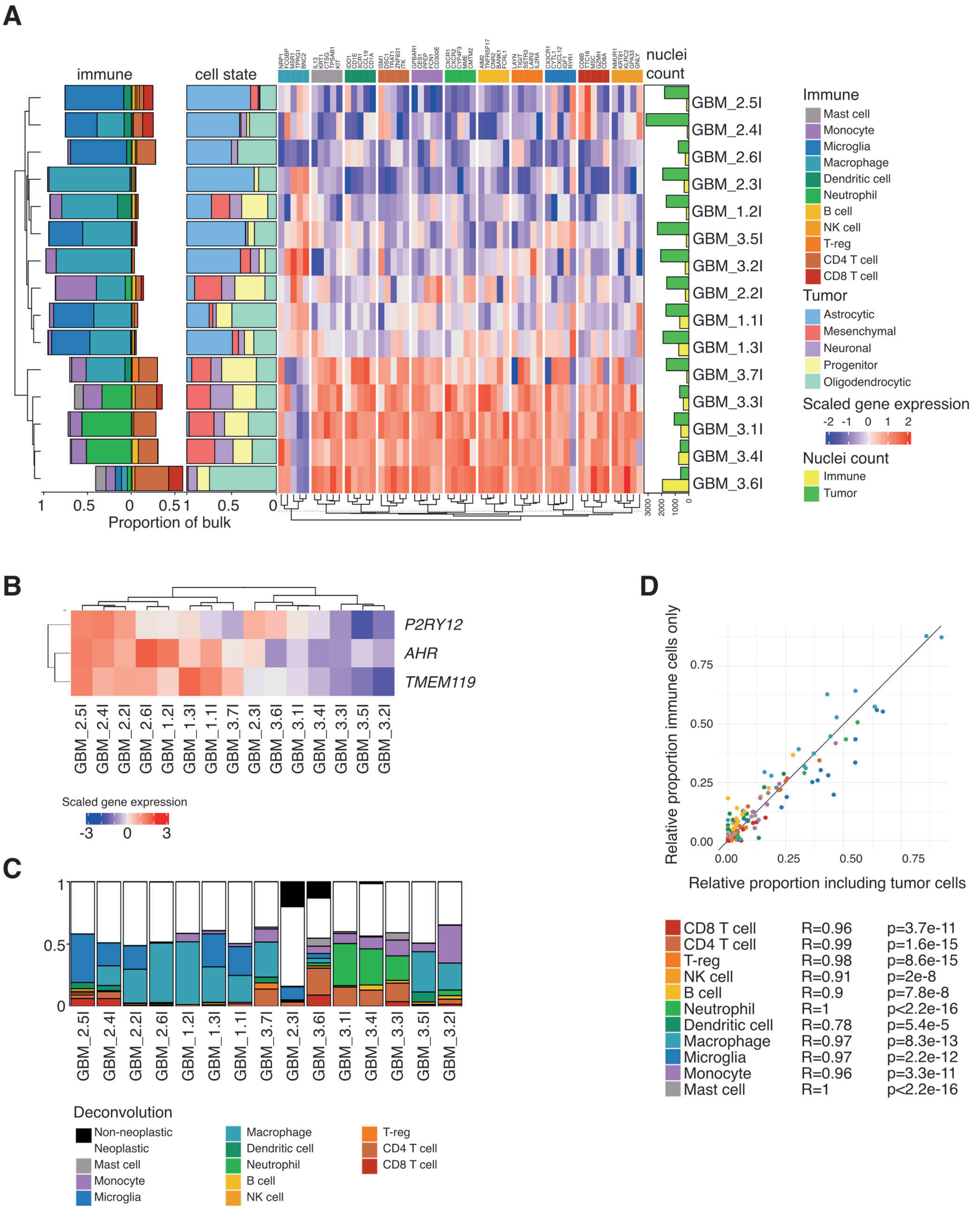

Supplementary Figure 3 |

A. Heatmap of immune cell type deconvolution signatures. Scaled gene expression of genes from Ruiz-Moreno *et al.*[6] dataset present in SafeTME reference and preprocessed gene list (n = 859), for each cell type profile. **B.** Expression of key macrophage and microglia genes *P2RY12, TMEM119* and *AHR* in *IDH1*-wt GBM immune samples. **C.** GBM Immune sample deconvolution including non-immune neoplastic and non-neoplastic cell types. **D.** Immune proportions pre and post tumor cell deconvolution. Below, correlations of proportions for each cell type.

Supplementary figure 4

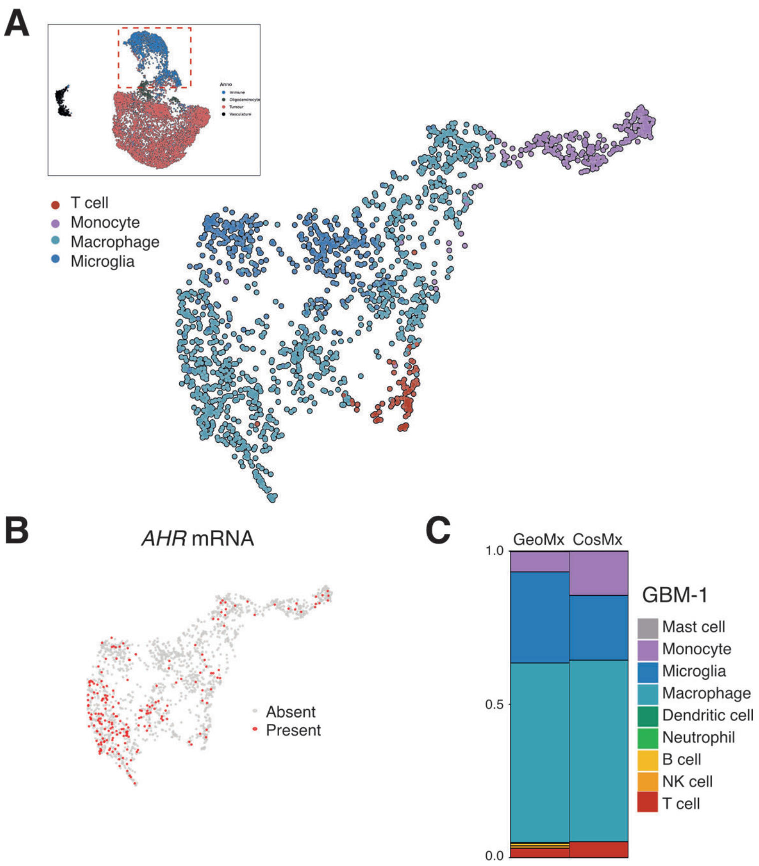

Supplementary Figure 4 |

A. UMAP of 1,847 immune cells colored by cell state type. Inset, UMAP of all cells with immune cluster indicated by red box. **B.** UMAP of immune cells indicating presence of AHR expression. **C.** Average immune infiltrate detected in GeoMx® compared to CosMx® analysis for GBM-1. All T cell populations in DSP analysis were combined into a single population to mirror the SMI populations.

Supplementary figure 5

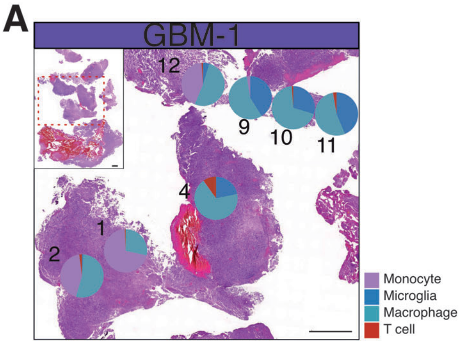

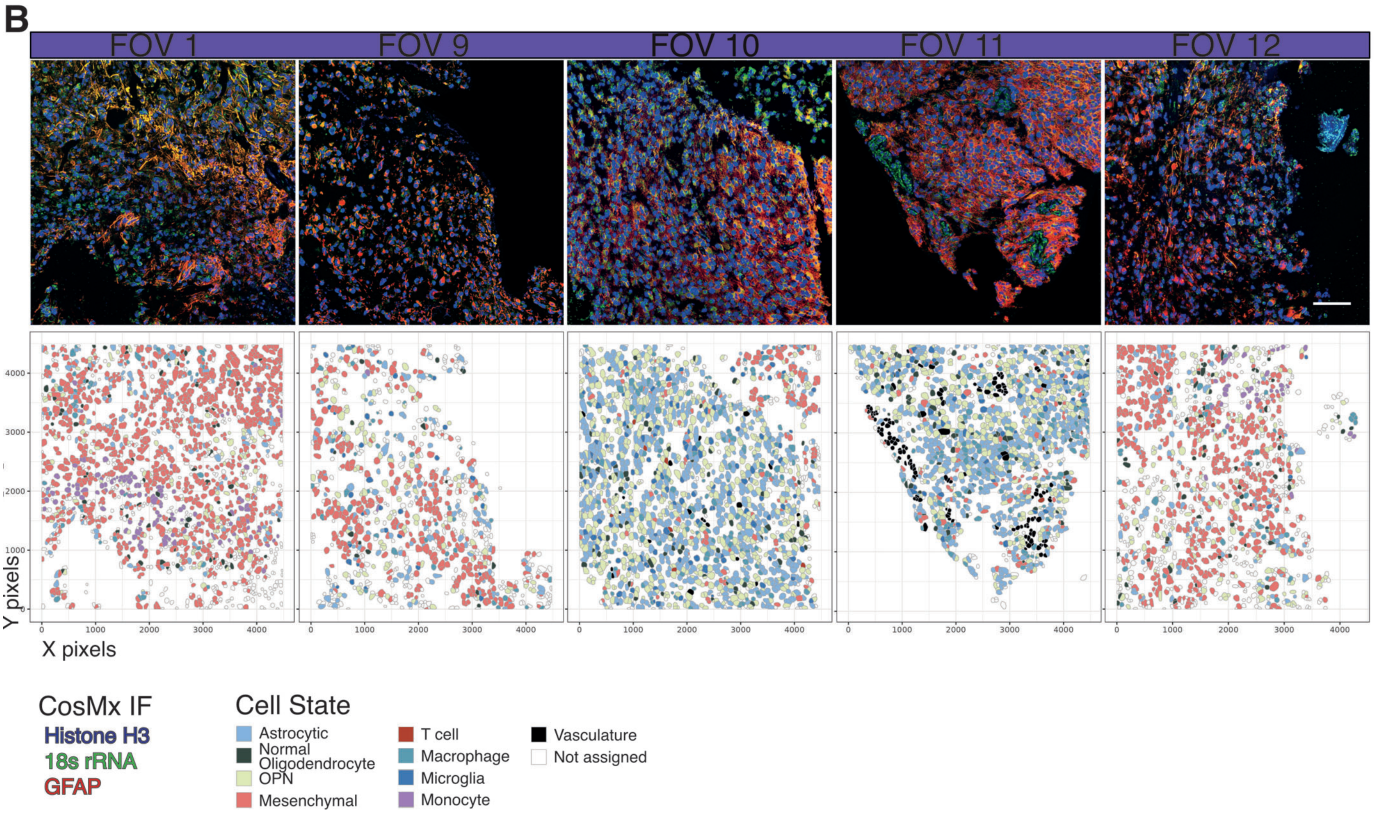

Supplementary Figure 5 |

A. H&E image of GBM-1 with proportions of immune cells plotted spatially in each analysis region. Scale, 1 mm. **B.** Immunofluorescence staining (Histone H3, 18s rRNA, GFAP) with corresponding Voronoi plots of each annotated cell state in GBM-1 regions (FOV: Field of View).

Supplementary figure 6

A

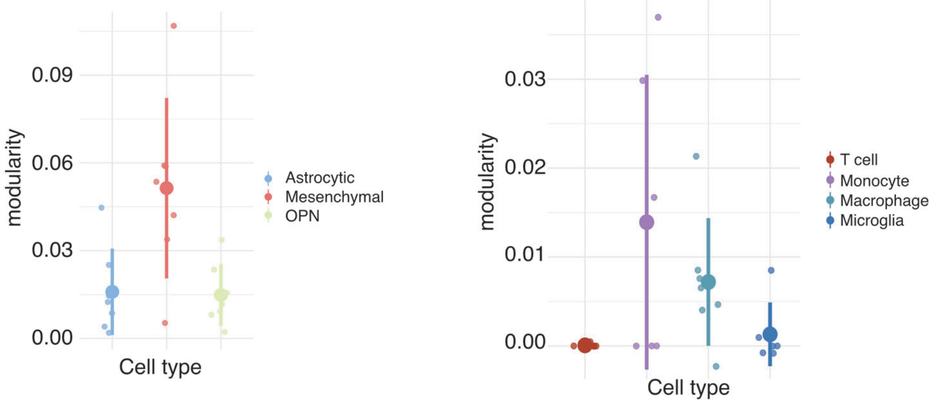

B

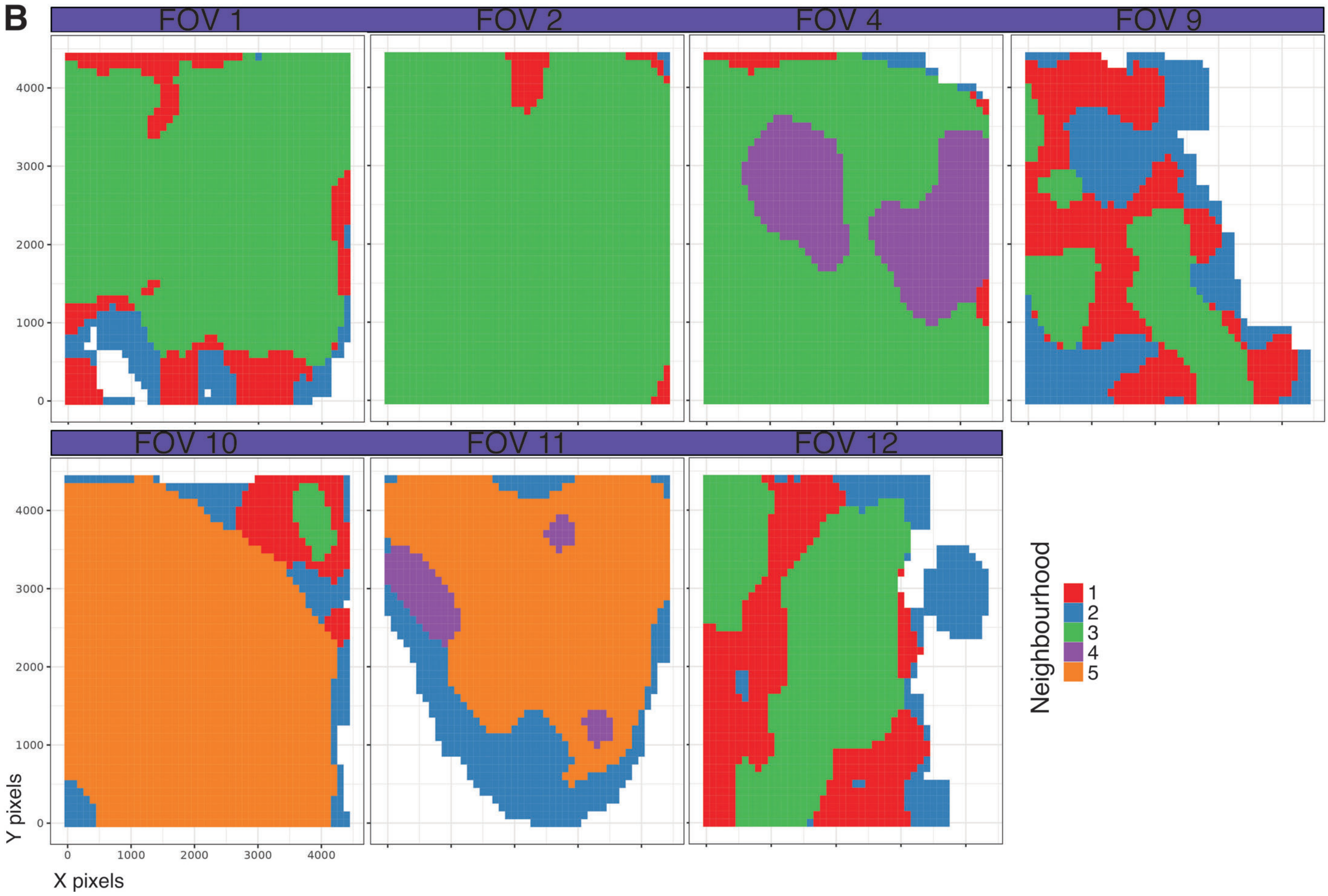

Supplementary Figure 6 |

A. Modularity score for tumor cell states (left) and immune cells (right) measuring the number of connections between cells of the same state compared to those of other states. **B.** Plot indicating neighborhoods for each region (FOV: Field of View) in GBM-1 single cell analysis. Scale, 100 μm.

Supplementary figure 7

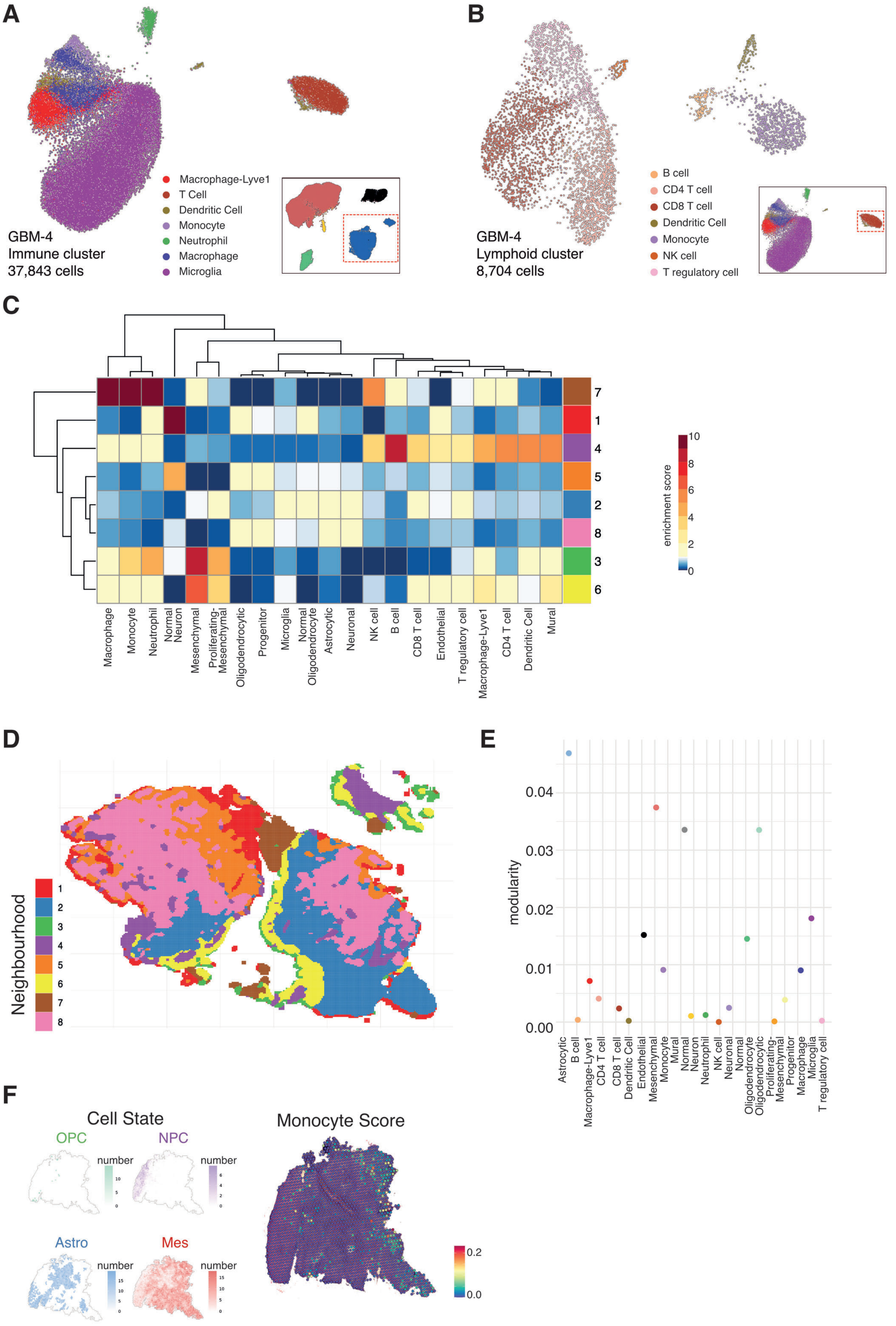

Supplementary Figure 7 |

A. UMAP of 37,843 immune cells colored by cell state type. Inset, UMAP of all cells with immune cluster indicated by red box. **B.** UMAP of 8,704 lymphoid cells colored by cell state type. Inset, UMAP of all immune with lymphoid cluster indicated by red box. **C.** Enrichment heatmap of each cell type identified co-localized within the neighborhoods in GBM-4. **D.** Plot indicating neighborhood locations across the tissue section of GBM-4. **E.** Modularity scores of each GBM-4 cell type measuring the number of connections between cells of the same state compared to those of other states. **F.** Representative GBM Visium® sample with spots colored by estimated number of cells of mesenchymal, NPC-like, OPC-like and astrocytic tumor state (left) and Monocyte score (right).

Supplementary figure 8

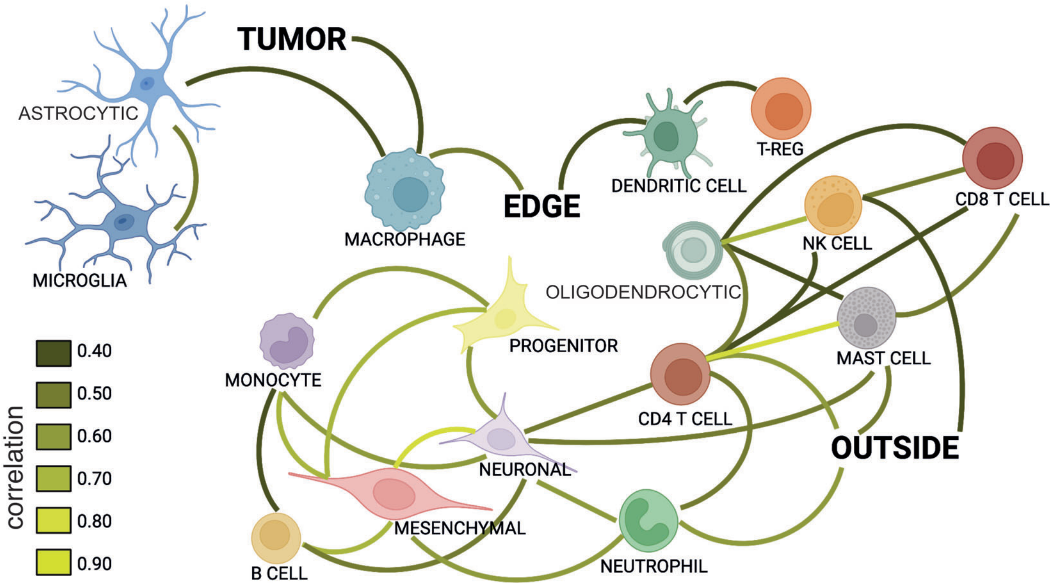

Supplementary Figure 8 |

Schematic of cell co-occurrence in the key domains of the tumor, edge and outside from GeoMx®
data. Line color relative to correlation coefficient.

Supplementary Table 1: *IDH1*-mutant samples area of interest (AOI) information

Sample	ROI	Pathology Review	Primary Classification	Secondary Classification	Immune Classification	AOI	Segment	Characteristic	Status
O_1	001	Cellular Tumor	Ki67 border	tumor	Low	GFAP+	tumor	Ki67+ region	Included
						CD45+	immune	Ki67+ region	Excluded
	002	Cellular Tumor	tumor		High	GFAP+Ki67+	cycling tumor	tumor	Included
						GFAP+	non-cycling tumor	tumor	Included
						CD45+	immune	tumor	Included
	003	Cellular Tumor	tumor		High	GFAP+Ki67+	cycling tumor	tumor	Included
						GFAP+	non-cycling tumor	tumor	Included
						CD45+	immune	tumor	Excluded
	004	Cellular Tumor	tumor		High	GFAP+	tumor	necrotic	Included
						CD45+	immune	necrotic	Included
	005	Cellular Tumor	Ki67 border		Low	GFAP+	tumor	Ki67- region	Included
						CD45+	immune	Ki67- region	Excluded
A_2	001	Cellular Tumor	Ki67 border	tumor	Low	GFAP+	tumor	Ki67+ region	Included
						CD45+	immune	Ki67+ region	Excluded
	002	Cellular Tumor	Ki67 border		Low	GFAP+	tumor	Ki67- region	Included
						CD45+	immune	Ki67- region	Included
	003	Cellular Tumor	border	tumor	Low	GFAP+	tumor	Ki67+ region	Included
						CD45+	immune	Ki67+ region	Excluded
	004	Infiltrating Tumor	border		Low	GFAP+	tumor	Ki67- region	Included
	005	Cellular Tumor	Ki67 border	tumor	Low	GFAP+	tumor	Ki67+ region	Included
						CD45+	immune	Ki67+ region	Included
	006	Cellular Tumor	tumor		High	GFAP+Ki67+	cycling tumor	tumor	Included
						GFAP+	non-cycling tumor	tumor	Included
						CD45+	immune	tumor	Included
A_3	001	Cellular Tumor	Ki67 border		Low	GFAP+	tumor	Ki67- region	Included
	002	Leading Edge	border		High	GFAP+Ki67+	cycling tumor	normal	Excluded
						GFAP+	non-cycling tumor	normal	Included
	003	Infiltrating Tumor	border		High	GFAP+Ki67+	cycling tumor	mix tumor	Included
						GFAP+	non-cycling tumor	mix tumor	Included
	004	Infiltrating Tumor	border		High	GFAP+Ki67+	cycling tumor	mix tumor	Included
						GFAP+	non-cycling tumor	mix tumor	Included
	005	Cellular Tumor	Ki67 border	tumor	Low	GFAP+	tumor	Ki67+ region	Included

Supplementary Table 2: *IDH*-wt samples area of interest (AOI) information

Sample	ROI	Pathology Review	Primary Classification	Secondary Classification	Immune Classification	AOI	Segment	Characteristic	Status
GBM_1	001	Cellular Tumor and Microvascular Proliferation	tumor		High	GFAP+Ki67+	cycling tumor	tumor	Included
						GFAP+	non-cycling tumor	tumor	Included
						CD45+	immune	tumor	Included
	002	Cellular Tumor	tumor		High	GFAP+Ki67+	cycling tumor	tumor	Included
						GFAP+	non-cycling tumor	tumor	Included
						CD45+	immune	tumor	Included
	003	Cellular Tumor	tumor		High	GFAP+Ki67+	cycling tumor	tumor	Included
						GFAP+	non-cycling tumor	tumor	Included
						CD45+	immune	tumor	Included
	004	Cellular Tumor	border	tumor	Low	GFAP+	tumor	tumor	Included
	005	Infiltrating Tumor	border		Low	GFAP+	tumor	mix tumor	Included
	006	Leading Edge	border		Low	GFAP+	tumor	normal	Included
GBM_2	002	Cellular Tumor	border		Low	GFAP+Ki67+	cycling tumor	tumor	Included
						GFAP+	non-cycling tumor	tumor	Included
						CD45+	tumor	tumor	Included
	003	Infiltrating Tumor	border		Low	GFAP+Ki67+	cycling tumor	normal	Excluded
						GFAP+	non-cycling tumor	normal	Included
						CD45+	tumor	normal	Included
	004	Cellular Tumor	Ki67 border	tumor	High	GFAP+	tumor	Ki67+ region	Included
						CD45+	immune	Ki67+ region	Included
	005	Cellular Tumor	Ki67 border		High	GFAP+	tumor	Ki67- region	Included
						CD45+	immune	Ki67- region	Included
	006	Cellular Tumor	border		Low	GFAP+Ki67+	cycling tumor	tumor	Included
						GFAP+	non-cycling tumor	tumor	Included
						CD45+	tumor	tumor	Included
GBM_3	001	Pseudo-palisading cells around Necrosis	Ki67 border		Low	GFAP+	tumor	Ki67- region	Included
						CD45+	immune	Ki67- region	Included
	002	Cellular Tumor	Ki67 border	tumor	Low	GFAP+	tumor	Ki67+ region	Included
						CD45+	immune	Ki67+ region	Included
	003	Perinecrotic Zone	Ki67 border		Low	GFAP+	tumor	Ki67- region	Included
						CD45+	immune	Ki67- region	Included
	004	Pseudo-palisading cells around Necrosis	Ki67 border		High	GFAP+	tumor	Ki67- region	Included
						CD45+	immune	Ki67- region	Included
	005	Pseudo-palisading cells around Necrosis	Ki67 border	tumor	High	GFAP+	tumor	Ki67+ region	Included
						CD45+	immune	Ki67+ region	Included
	006	Necrosis	border		High	GFAP+	tumor	normal	Included
						CD45+	immune	normal	Included
	007	Cellular Tumor	Ki67 border	tumor	Low	GFAP+	tumor	Ki67+ region	Included
						CD45+	immune	Ki67+ region	Included

TABLE S3 | Quantification of immune cell infiltration patterns in glioblastoma cohort

Sample	IHC stain	Topographic Regions		
		Tumor parenchyma	Perivascular space	Necrosis
GBM-1	CD45	1% – 2%	1% – 2%	0%
	CD3	2% – 3%	3% – 4%	0%
	CD68	5% – 10%	3% – 5%	10% – 40%
GBM-2	CD45	13% – 21%	22% – 35%	35% – 50%
	CD3	3% – 8%	11% – 15%	10% – 16%
	CD68	5% – 7%	15% – 18%	25% – 35%
GBM-3	CD45	35% – 55%	40% – 60%	0%
	CD3	6% – 9%	2% – 4%	0%
	CD68	10% – 15%	2% – 5%	0%

FIGURE S4 | CosMx® SMI 1,000 gene panel

AATK	BTG1	CENPF	DDC	FZD6	IGF2	KRAS	NKG7	RGCC	STAT1	VWF
ABL1	BTK	CFD	DDIT3	FZD7	IGF2R	KRT1	NLRC4	RGS1	STAT3	WIF1
ABL2	C11orf96	CFLAR	DDR1	FZD8	IGFBP3	KRT10	NLRC5	RGS2	STAT4	WNT10B
ACE	C1QA	CHEK1	DDR2	G6PC2	IGFBP5	KRT13	NLRP1	RGS5	STAT5A	WNT11
ACE2	C1QB	CHEK2	DDX58	G6PD	IGFBP6	KRT14	NLRP12	RNF43	STAT5B	WNT2
ACKR1	C1QC	CHGA	DHRS2	GADD45B	IGFBP7	KRT15	NLRP2	ROR1	STAT6	WNT2B
ACKR3	C5AR2	CHI3L1	DIO2	GAPDH	IGHA1	KRT16	NLRP3	RORA	STMN1	WNT3
ACKR4	C9orf16	CIDEA	DLL1	GAS6	IGHD	KRT17	NOD2	RPL10	SUCNR1	WNT5A
ACTA2	CALB1	CIITA	DMBT1	GATA3	IGHG1	KRT18	NOSIP	RPL18A	SYK	WNT5B
ACTB	CALD1	CLCF1	DNMT1	GC	IGHG2	KRT19	NOTCH1	RPL19	TACSTD2	WNT7A
ACTG1	CALM1	CLDN4	DNMT3A	GCG	IGHM	KRT20	NOTCH2	RPL21	TAGLN	WNT7B
ACTG2	CALM2	CLEC10A	DNTT	GDF10	IGKC	KRT222	NOTCH3	RPL22	TAP1	WNT9A
ACVR1	CALM3	CLEC12A	DPP4	GDF11	IL10	KRT23	NPPB	RPL26	TAP2	XBP1
ACVR1B	CAMP	CLEC14A	DST	GDF15	IL10RA	KRT24	NPPC	RPL28	TBX21	XCL1
ACVR2A	CASP3	CLEC1A	DUSP1	GDF3	IL10RB	KRT4	NPR1	RPL32	TCL1A	XCL2
ACVRL1	CASP8	CLEC2B	DUSP2	GDF6	IL11	KRT5	NPR2	RPL34	TEK	YBX3
ADGRA2	CASR	CLEC2D	DUSP4	GDF9	IL11RA	KRT6A	NPR3	RPL37	TFEB	YES1
ADGRA3	CAV1	CLEC4A	DUSP5	GDNF	IL12A	KRT6B	NR1H2	RPL39	TGFB1	ZFP36
ADGRB2	CCL11	CLEC4D	DUSP6	GLUD1	IL12B	KRT6C	NR1H3	RPL7	TGFB2	
ADGRB3	CCL13	CLEC4E	EEF1A1	GLUL	IL12RB1	KRT7	NR1H4	RPLP1	TGFB3	
ADGRD1	CCL15	CLEC5A	EFNA1	GNLY	IL12RB2	KRT8	NR3C1	RPLP2	TGFBR1	
ADGRE1	CCL18	CLEC7A	EFNA4	GPBAR1	IL13RA1	KRT80	NRG1	RPS12	TGFBR2	
ADGRE2	CCL19	CLOCK	EFNA5	GPER1	IL15	KRT86	NRG4	RPS14	THBS1	
ADGRE5	CCL2	CLU	EFNB1	GPNMB	IL15RA	LAG3	NRIP3	RPS15A	THBS2	
ADGRF1	CCL20	CMKLR1	EFNB2	GPR183	IL16	LAIR1	NRXN1	RPS18	TIE1	
ADGRF3	CCL21	CNTFR	EFNB3	GPX1	IL17A	LAMP2	NRXN3	RPS19	TIGIT	
ADGRF4	CCL23	COL11A1	EGF	GPX3	IL17B	LAMP3	NTRK2	RPS21	TIMP1	
ADGRF5	CCL26	COL12A1	EGFR	GSN	IL17D	LCN2	OLFM4	RPS23	TLR1	
ADGRG1	CCL28	COL14A1	EIF5A	GSTP1	IL17RA	LDLR	OLR1	RPS24	TLR2	
ADGRG2	CCL3	COL15A1	ELANE	GUCA2A	IL17RB	LEFTY1	OSM	RPS27	TLR3	
ADGRG3	CCL3L3	COL16A1	EMP3	GUCY2C	IL17RE	LEFTY2	OSMR	RPS28	TLR4	
ADGRG5	CCL4	COL17A1	ENG	GZMA	IL18	LEP	OXER1	RPS3A	TLR5	
ADGRG6	CCL4L2	COL18A1	ENSG00000257764	GZMB	IL18R1	LGALS1	OXGR1	RPS4X	TLR7	
ADGRL1	CCL5	COL1A1	ENSG00000258017	GZMH	IL1A	LGALS3	P2RX5	RPS4Y1	TLR8	
ADGRL2	CCL7	COL1A2	ENSG00000264577	GZMK	IL1B	LGALS3BP	P2RY12	RPS5	TM4SF1	
ADGRL4	CCL8	COL21A1	ENSG00000269968	H2AZ1	IL1R1	LGALS9	PARP1	RPS6	TMSB10	
ADGRV1	CCND1	COL27A1	ENTPD1	H4C3	IL1R2	LIF	PCNA	RPS8	TMSB4X	
ADIPOQ	CCR1	COL3A1	EOMES	HAVCR2	IL1RAP	LIFR	PDCD1	RSPO1	TNF	
ADIRF	CCR10	COL4A1	EPCAM	HBA1	IL1RL1	LINC02446	PDCD1LG2	RSPO2	TNFAIP6	
ADM2	CCR2	COL4A2	EPHA2	HBB	IL1RN	LMNA	PDGFA	RSPO3	TNFRSF10A	
ADORA2A	CCR5	COL4A5	EPHA3	HCAR2	IL2	LPAR5	PDGFB	RUNX3	TNFRSF10B	
AGR2	CCR7	COL5A1	EPHA4	HCAR3	IL20	LTB	PDGFC	RXRA	TNFRSF10D	
AHI1	CCRL2	COL5A2	EPHA7	HCK	IL20RA	LTBR	PDGFD	RXRB	TNFRSF11A	
AHR	CD14	COL5A3	EPHB2	HCST	IL22RA1	LTF	PDGFRA	RYK	TNFRSF11B	
AKT1	CD163	COL6A1	EPHB3	HDAC1	IL23A	LUM	PDGFRB	S100A10	TNFRSF12A	
ALCAM	CD164	COL6A2	EPHB4	HDAC11	IL24	LY6D	PECAM1	S100A2	TNFRSF13B	
AMHR2	CD19	COL6A3	EPHB6	HDAC3	IL27RA	LY75	PF4	S100A4	TNFRSF14	
ANGPT1	CD2	COL8A1	EPOR	HDAC4	IL2RA	LYN	PGF	S100A6	TNFRSF17	
ANGPT2	CD209	COL8A2	ERBB2	HDAC5	IL2RB	LYZ	PGR	S100A8	TNFRSF18	
ANGPT4	CD24	COL9A1	ERBB3	HEBP1	IL2RG	MAF	PHLDA2	S100A9	TNFRSF19	
ANGPTL1	CD248	COL9A2	ESAM	HGF	IL32	MALAT1	PIGR	S100B	TNFRSF1A	
ANXA1	CD27	COL9A3	ESR1	HIF1A	IL33	MAML2	PLA2R1	S100P	TNFRSF1B	
ANXA2	CD274	COPA	ETS1	HILPDA	IL34	MAP1LC3B	PLAC8	SAA1	TNFRSF21	
ANXA4	CD276	COTL1	ETV4	HLA-A	IL36G	MAPK13	PNOC	SAA2	TNFRSF4	
APOA1	CD28	CPA3	ETV5	HLA-B	IL3RA	MAPK14	POU5F1	SAT1	TNFRSF9	
APOB	CD300A	CPB1	EZH2	HLA-C	IL4R	MARCO	PPARA	SCG5	TNFSF10	
APOD	CD33	CRIP1	EZR	HLA-DPA1	IL6	MECOM	PPARD	SCGB3A1	TNFSF12	
APP	CD34	CRP	FABP4	HLA-DPB1	IL6R	MEG3	PPARG	SEC23A	TNFSF13B	
AQP3	CD36	CRYAB	FABP5	HLA-DQA1	IL6ST	MERTK	PPBP	SEC24A	TNFSF14	
AR	CD37	CSF1	FAS	HLA-DQB1	IL7	MET	PRF1	SEC61G	TNFSF15	
AREG	CD38	CSF1R	FASLG	HLA-DRA	IL7R	MGP	PROK2	SELENOP	TNFSF18	
ARF1	CD3D	CSF2	FASN	HLA-DRB1	INHA	MIF	PROKR1	SELL	TNFSF4	
ARG1	CD3E	CSF2RA	FCER1G	HLA-DRB5	INHBA	MKI67	PRSS2	SELPLG	TNFSF8	
ARHGDIB	CD3G	CSF2RB	FCGBP	HLA-E	INHBB	MMP1	PSAP	SERPINA1	TNFSF9	
ARTN	CD4	CSF3	FCGR3A	HMGB2	INS	MMP10	PSCA	SERPINA3	TNK1	
ATF3	CD40	CSF3R	FCRLA	HMGN2	INSR	MMP12	PSPN	SERPINB5	TOP2A	
ATG10	CD40LG	CSHL1	FES	HPGDS	IRF4	MMP14	PTGDR2	SERPINH1	TOX	
ATG12	CD44	CSK	FFAR2	HSD17B2	ITGA1	MMP15	PTGDS	SFN	TP53	

ATG5	CD47	CST7	FFAR3	HSD3B2	ITGA2	MMP16	PTGES	SIGIRR	TPM1	
ATM	CD48	CTLA4	FFAR4	HSP90AA1	ITGA3	MMP19	PTGES2	SLC2A1	TPM2	
ATR	CD52	CTNNB1	FGF1	HSP90AB1	ITGA5	MMP2	PTGES3	SLC2A4	TPSAB1	
AXL	CD53	CTSG	FGF12	HSP90B1	ITGA6	MMP3	PTGIS	SLC40A1	TPSB2	
AZGP1	CD55	CTSW	FGF13	HSPA1A	ITGA9	MMP7	PTGS1	SLPI	TSC22D1	
AZU1	CD58	CUZD1	FGF18	HSPA1B	ITGAE	MMP8	PTGS2	SMAD2	TSHZ2	
B2M	CD59	CX3CL1	FGF2	HSPB1	ITGAL	MMP9	PTHLH	SMAD3	TSLP	
B3GNT7	CD5L	CX3CR1	FGF7	HTT	ITGAM	MPO	PTK2	SMAD4	TTR	
BAG3	CD63	CXCL1	FGF9	IAPP	ITGAV	MRC1	PTK6	SMARCB1	TUBA1B	
BATF3	CD68	CXCL10	FGFR1	ICAM1	ITGAX	MRC2	PTK7	SMO	TUBB	
BAX	CD69	CXCL12	FGFR2	ICAM2	ITGB1	MS4A1	PTN	SNAI1	TUBB4B	
BCL2	CD70	CXCL14	FGFR3	ICAM3	ITGB2	MS4A4A	PTPRC	SNAI2	TWIST1	
BCL2L1	CD74	CXCL16	FGG	ICOS	ITGB4	MSMB	PTPRCAP	SOD1	TWIST2	
BECN1	CD79A	CXCL17	FGR	ICOSLG	ITGB5	MST1R	PTTG1	SOD2	TXK	
BEST1	CD80	CXCL2	FKBP11	IDO1	ITGB6	MT1X	QRFPR	SOSTDC1	TYK2	
BGN	CD81	CXCL3	FLT1	IER3	ITGB8	MT2A	RAC1	SOX2	TYMS	
BID	CD83	CXCL5	FLT3LG	IFI27	ITK	MTOR	RAC2	SOX4	TYROBP	
BIRC5	CD84	CXCL6	FN1	IFIH1	ITM2A	MTRNR2L1	RAD51	SOX9	UBE2C	
BMP1	CD86	CXCL8	FOS	IFITM1	JAG1	MXRA8	RAMP1	SPARCL1	UCP1	
BMP2	CD8A	CXCL9	FOXF1	IFITM3	JAK1	MYC	RAMP2	SPINK1	UPK3A	
BMP3	CD8B	CXCR1	FOXP3	IFNA1	JAK2	MYH11	RAMP3	SPOCK2	VCAM1	
BMP4	CD9	CXCR2	FPR1	IFNAR1	JCHAIN	MYL9	RARA	SPP1	VCAN	
BMP5	CDH1	CXCR3	FRK	IFNAR2	JUN	MZB1	RARB	SPRY2	VEGFA	
BMP6	CDH11	CXCR4	FTH1	IFNB1	JUNB	MZT2A	RARG	SPRY4	VEGFB	
BMP7	CDH5	CXCR5	FTL	IFNG	KDR	NANOG	RARRES1	SQSTM1	VEGFC	
BMPR1A	CDKN1A	CXCR6	FYB1	IFNGR1	KIT	NCR1	RARRES2	SRC	VEGFD	
BMPR2	CDKN3	CYP19A1	FYN	IFNGR2	KITLG	NDRG1	RB1	SREBF1	VHL	
BMX	CEACAM1	CYP1B1	FZD1	IFNL2	KLF2	NEAT1	RBPJ	SRGN	VIM	
BRCA1	CEACAM6	CYSTM1	FZD3	IFNL3	KLK3	NFKB1	REG1A	SST	VPREB3	
BST1	CELSR1	CYTOR	FZD4	IGF1	KLRB1	NFKBIA	RELA	ST6GAL1	VSIR	
BST2	CELSR2	DCN	FZD5	IGF1R	KLRK1	NGFR	RELT	ST6GALNAC3	VTN	

TABLE S5 | Xenium® Gene Panel

AATK	CD68	EPHA4	HLA-DQB1	MAG	PDZRN3	SPARC
ABCA2	CD69	EPHB1	HLA-DRB5	MAL	PECAM1	SPI1
ABCC3	CD7	ERBB3	HMOX1	MAML2	PGM2	SPOCK3
AIF1	CD79A	ERMN	HOPX	MAP1B	PGM3	SRI
AKAP12	CD79B	ETV1	HOXD3	MAP2	PLIN2	STAT3
ALOX5AP	CD83	FABP6	HPCA	MBP	PMP2	STMN2
ANXA1	CD86	FABP7	HS3ST4	MDM2	POSTN	STXBP2
APOD	CD8A	FASLG	IDH1	MEF2C	PRDX2	SULT1A3
APOE	CD8B	FBLN1	IDH2	MEIS2	PROX1	TENM3
AQP4	CDH12	FBXO36	IDO1	MEOX2	PTCHD4	TGFB1
AREG	CDH13	FCER1A	IDO2	MEST	PTEN	TGFB2
ARHGDIB	CDH4	FCER1G	IER3	METRN	PTPRA	TGFBI
ASNS	CDK1	FCGBP	IFI27	MGAT4C	PTPRC	TMIGD3
B4GALNT1	CDK4	FCGR1A	IGFBP3	MGST1	PTPRK	TNFAIP3
BANK1	CDKN1C	FCGR3A	IGFBP4	MKI67	PTPRT	TOP2A
BCAN	CDKN2A	FGFR2	IGFBP5	MMP7	PTPRZ1	TRAC
BCHE	CENPF	FGFR3	IGFBP7	MOBP	RAB3C	TRBC1
BCL3	CH25H	FOXJ1	IL13RA2	MOG	RACGAP1	TRBC2
BIN1	CHCHD7	FOXP3	IL1B	MRPS17	RAPGEF5	TREM2
BRINP3	CHI3L1	G0S2	IL1RAPL2	MS4A6A	RASGEF1B	TRIL
C15orf48	CLDN11	GABRB3	IL2RA	MT1G	RB1	TRPM3
C1QL3	CNDP1	GAP43	IL32	MT1H	RBFOX3	TRPS1
C1orf162	CNN3	GAPDH	IL6	NF1	RBP1	TSC22D1
C1orf194	CNTN2	GATM	IL7R	NFIA	RELN	TSHZ2
C9orf24	CNTN5	GCK	ITGAM	NKAIN3	RFX3	TSPAN3
CA10	COL1A2	GFPT1	ITGAX	NKG7	RGS10	TTYH1
CAPG	CORO1A	GFPT2	ITGB1	NMB	RGS4	TUBB2B
CAPN3	COTL1	GJA1	ITGB2	NNAT	RGS6	TYMP
CAV1	CSPG4	GLYAT	ITGB8	NPDC1	RNASET2	UGT1A1
CCL20	CST3	GMPS	KCNMB4	NPTXR	ROBO1	UGT8
CCL5	CSTA	GNLY	KIRREL3	NR4A2	RORB	VAMP8
CCNA1	CTLA4	GPC6	KISS1R	NRGN	S100A1	VCAN
CCNB2	CTSH	GPI	KIT	NRN1	S100A12	VDR
CCR7	CTSS	GPNMB	KLF2	NRP1	S100A16	VGF
CD14	CX3CR1	GPR183	KLF4	NT5C	S100A4	VSIG4
CD163	CXCR1	GPR34	KLK6	NT5M	SAA1	WIPF3
CD19	CXCR4	GRIK1	KLRB1	NTNG1	SCRG1	ZBTB20
CD1A	CYP1A2	GRM8	KLRC1	NTRK2	SEPTIN11	ZMAT4
CD1C	CYTIP	GZMB	LANCL2	NUPR1	SFRP2	ZNF804B
CD2	DCC	HAMP	LHPP	NWD2	SGCD	
CD247	DCN	HAT1	LIFR	OLIG1	SGCZ	
CD27	DLL3	HDAC2	LILRA4	OLIG2	SHISA6	
CD274	DNER	HEPN1	LMO4	OLIG3	SLC11A1	
CD28	DUSP2	HES6	LOX	OTOS	SLC24A2	
CD3D	EDIL3	HHATL	LPL	P2RY12	SLPI	
CD3G	EFHD1	HILPDA	LST1	P2RY13	SOX10	
CD4	EGFR	HK1	LTB	PAX6	SOX11	
CD40	ELOVL2	HK2	LY86	PCNA	SOX2	
CD48	ENPP1	HLA-DMB	LYVE1	PDCD1	SOX4	
CD52	EPCAM	HLA-DQA1	MAF	PDGFRA	SOX9	
